# Facile one-pot reduction of β-nitrostyrenes to phenethylamines using sodium borohydride and copper(II) chloride

**DOI:** 10.3762/bjoc.21.4

**Published:** 2025-01-07

**Authors:** Laura D’Andrea, Simon Jademyr

**Affiliations:** 1 Department of Drug Design and Pharmacology, Faculty of Health and Medical Sciences, University of Copenhagen, Universitetsparken 2, 2100 København Ø, Denmark; 2 current address: Department of Chemistry and Bioscience, Aalborg University, Fredrik Bajers Vej 7H, 9220 Aalborg, Denmark; 3 current address: Centre for Analysis and Synthesis, Lund University, Naturvetarvägen 14, 223 62 Lund, Sweden

**Keywords:** 2C-X, CuCl_2_, NaBH_4_, β-nitrostyrene, phenethylamine

## Abstract

Phenethylamines and phenylisopropylamines of scientific relevance can be prepared with a NaBH_4_/CuCl_2_ system in 10 to 30 minutes via reduction of substituted β-nitrostyrenes. This one-pot procedure allows the quick isolation of substituted β-nitrostyrene scaffolds with 62–83% yield under mild conditions, without the need for special precautions, inert atmosphere, and time-consuming purification techniques.

## Introduction

The phenethylamine scaffold represents a recurring motif among natural and synthetic drug molecules. The latter are mainly constituted by a varied class of substituted phenylethylamines exhibiting psychoactive properties, and typically employed for medical and recreational use [1–2]. Representative examples include CNS stimulants (amphetamine), antidepressants and antiparkinson’s agents (e.g., ʟ-deprenyl) [3], hallucinogens and entactogens (e.g., 2,5-dimethoxy-4-iodoamphetamine (DOI) and 3,4-methylenedioxy-*N*-methylamphetamine (MDMA)) [4–5], nasal decongestants (e.g., levomethamphetamine), and appetite suppressants (e.g., phentermine) [6].

Phenethylamines can be produced via numerous different procedures [7]. One of the oldest methods involves the reduction of benzyl cyanide with H_2_ in liquid ammonia with Raney-Nickel catalyst at 130 °C, and high pressure [8]. Another known method is based on the reductive amination of phenyl-2-propanone by use of Al/Hg amalgam. The latter procedure involves numerous drawbacks, such as environmental concerns for the use of mercury, contamination of the final products, the need of special safety precautions, and adequate disposal techniques [9–10].

**Scheme 1:**
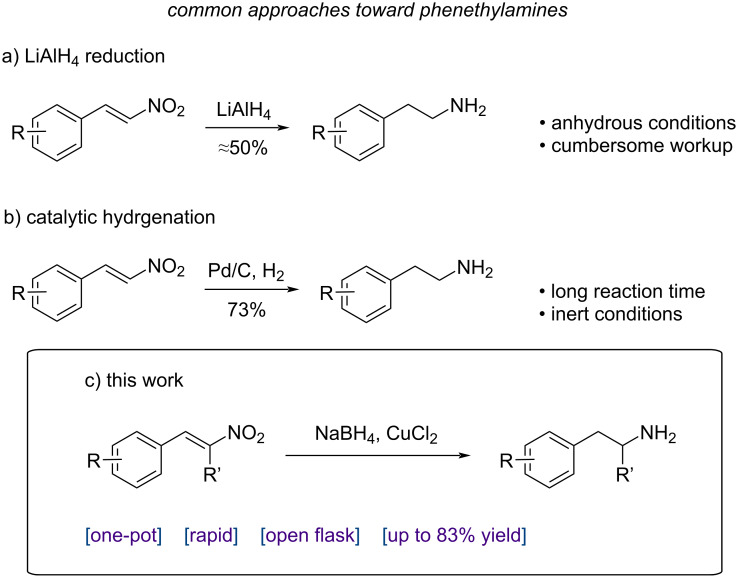
Brief comparison between the main traditional synthetic routes for the preparation of substituted phenethylamines from β-nitrostyrene scaffolds and our work.

One of the most studied and inexpensive routes to synthesize substituted phenethylamines focuses on the reduction of their α,β-unsaturated nitroalkene analogue (β-nitrostyrene), where both the double bond and the nitro group need to be reduced to deliver the corresponding primary amine. Their reduction can be accomplished via catalytic hydrogenation, involving stepwise reactions and workup, use of additional reagents, and reaction time between 3 and 24 hours [11–12]. Most commonly, metal hydrides are employed, typically lithium aluminum hydride [13–18], requiring an inert atmosphere, special precautions, and with isolated yields up to 60% [14–15]. Due to the formation of side products, final purification of the amino derivatives requires the use of either multiple separation techniques, chromatography, or distillation [15–18] (Scheme 1).

Differently from lithium aluminum hydride, sodium borohydride is a non-pyrophoric and easy-to-handle reducing agent. Since the first attempts in 1967, NaBH_4_ has been employed to reduce β-nitrostyrene scaffolds to the corresponding nitroalkanes [19–21]. Several catalysts have been combined with NaBH_4_ to facilitate full reduction of β-nitrostyrenes to phenethylamines, however, to date, no effective method for converting α,β-unsaturated nitroalkenes into aminoalkanes have been developed using NaBH_4_ as reducing agent [21–22].

The number of procedures reported in the literature regarding the reduction of β-nitrostyrenes is limited, since a NaBH_4_/transition metal salt system is mostly used to reduce nitroarenes [23–26].

One of the reported methods takes advantage of titanium(IV) isopropoxide as a catalyst to prepare varied β-phenethylamine analogues. Despite its simplicity, the reaction time is quite prolonged (from 18 to 20 hours), and this procedure is not used to prepare α-phenethylamines [27].

**Figure 1:**
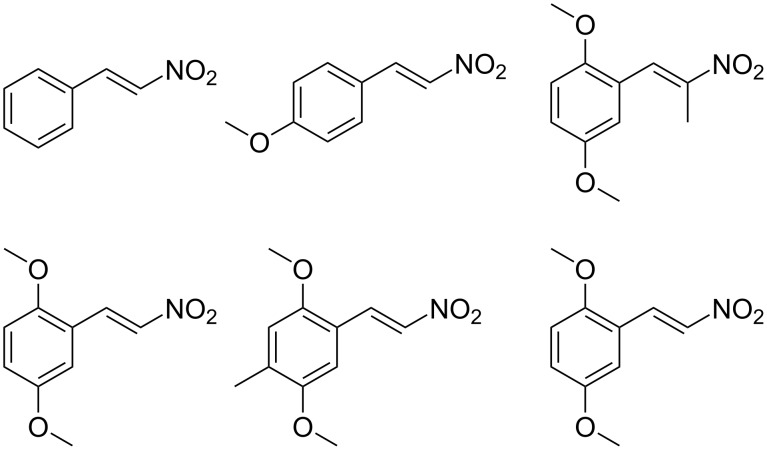
The β-nitrostyrene analogues used in this work.

In view of the limitations associated with conventional methods, we report our findings on an improved approach for reducing β-nitrostyrenes to their corresponding substituted phenethylamines. We demonstrate that the NaBH_4_/CuCl_2_ system effectively facilitates this transformation and provide an account of its application to the β-nitrostyrene examples presented in Figure 1.

## Result and Discussion

Herein, we demonstrate that NaBH_4_, in combination with catalytic amounts of CuCl_2_, is a simple and higher yielding method to synthesize phenethyl- and phenylisopropylamines from the corresponding nitroalkenes [15,17]. Representatively substituted β-nitrostyrene analogues were reduced via this method at 80 ˚C, including 2,5-dimethoxy-β-methyl-β-nitrostyrene (**3a**), precursor of amphetamines, and 2,5-dimethoxy-β-nitrostyrene (**4a**), precursor of most of the hallucinogenic 2C-X family (Table 1).

**Table 1:** The reduced β-nitrostyrene scaffolds with their corresponding products (entries 1–6). The isolated product yields were obtained by performing the reactions at 80 °C for the time indicated beside each product. For more details, see the experimental section below.

Entry	Substrate	Product	Time (min)	Yield (%)

1	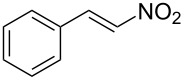 **1a**	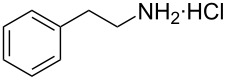 **1b**	15	83
2	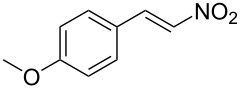 **2a**	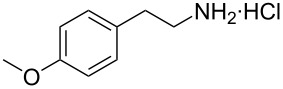 **2b**	10	82
3	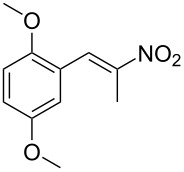 **3a**	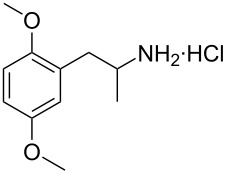 **3b**	30	62
4	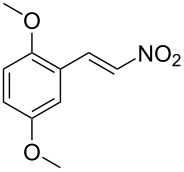 **4a**	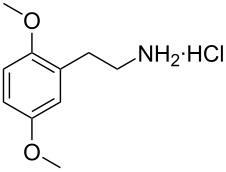 **4b**	10	82
5	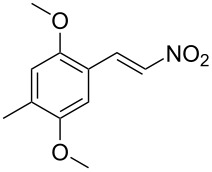 **5a**	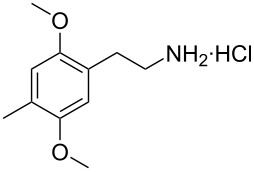 **5b**	30	65
6	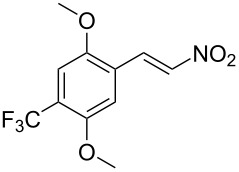 **6a**	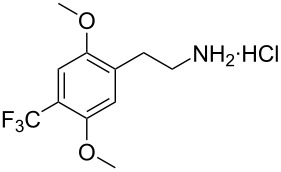 **6b**	30	71

**Scheme 2:**
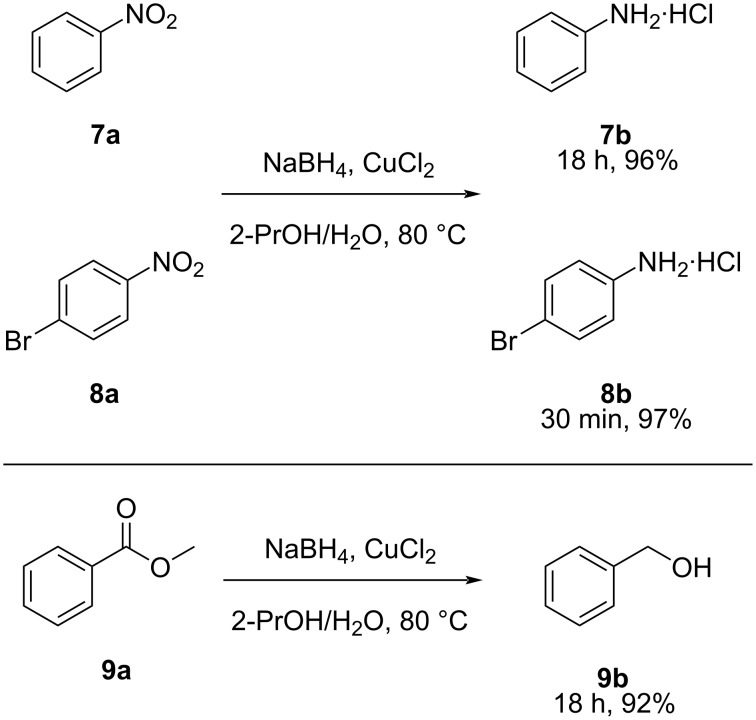
Additional products obtained via this method: nitrobenzene and methyl benzoate are reduced in excellent yields.

This method was also tested on other types of scaffolds to investigate its potential general applications and effects on other substituents. As sodium borohydride per se does not reduce ester nor nitro functionalities [15–22,28–29], the presence of the copper salt results in overcoming this issue and leads to isolated yields above 90% (**7**–**9**) (Scheme 2).

Therefore, the NaBH_4_/CuCl_2_ system was proved to work on aromatic ester, nitro, and α,β-unsaturated nitroalkene functionalities.

Our work demonstrates that, up to 24 hours, this method shows some degree of functional group tolerance, as the amido and carboxylic acid functionalities of benzamide and benzoic acid, were left untouched, and the starting materials were finally fully recovered.

1-Bromo-4-nitrobenzene (**8a**) and 3-chlorophenol were used to test the potential effects on halogenated aromatic structures and no dehalogenation was detected up to 24 hours stirring. The retention of halogen atoms on aryl halides distinguishes this procedure from traditional techniques, such as those involving LiAlH_4_, which can cause dehalogenation [30–31].

The role of the CuCl_2_ salt is pivotal to the success of this method. Studies on the reduction of CuCl_2_ by NaBH_4_ suggest that copper(II) is promptly reduced to free Cu(0), composing up to 96% of the products. The remaining 4% consist of Cu_2_O and negligible amounts of other copper species [32–33]. Consistently, once the chloride is added, the reduction to free Cu(0) is visually indicated by the immediate disappearance of the blue color of the copper(II) solution, and the formation of a fine suspended black powder. The latter, as metallic copper particles, acts as the actual catalyst.

Time plays a crucial role in the synthesis of phenethylamine analogues via this method. Dithering before the addition of the copper solution leads to the formation of Micheal adducts, which decrease the product yields. This phenomenon is due to the nature of β-nitrostyrenes, displaying considerable delocalization towards the nitro group, which makes them highly susceptible to Michael addition [34].

While being stirred with the borohydride, the substrate progressively forms an α-carbanion in the newly formed nitroalkane, which ultimately leads to Michael addition to the nitrostyrene.

Furthermore, studies to identify the highest yielding reaction times (reaction stopped at 10, 15, 30, 45, 60, 75, and 90 minutes) revealed that longer stirring when heating is applied is not beneficial. In general, soon after the optimal reaction times indicated in Table 1, the yield progressively decreases when the reaction stirring time increases. MS analyses on **4b** showed consistently that, while the product mass decreases over time, high molecular mass compounds form increasingly (MS data for **4b** can be found in Supporting Information File 1).

**Figure 2:**
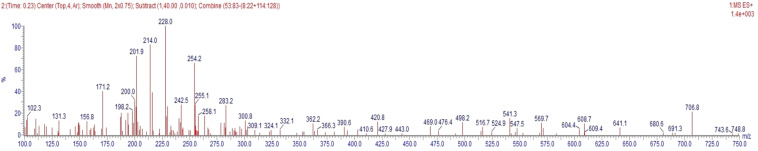
Numerous masses (*m*/*z*) were detected by ESI-MS at *T* = 0 upon mixing all the reagents to produce **1b**.

Over the course of the reaction to form **1b**, MS analyses indicated the prompt formation of numerous intermediate species at *T* = 0, unstable enough to decompose and deliver the desired product (Figure 2).

**Figure 3:**
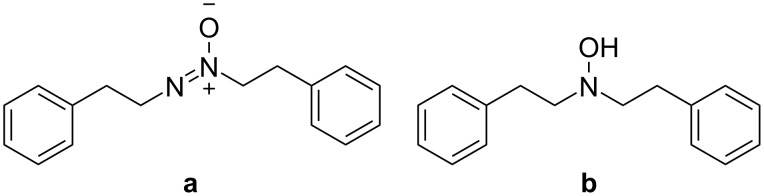
Structures of proposed adducts. Their masses, 254.2 and 242.2, respectively, were found at *T* = 0 by UPLC-MS investigation.

These species were not present in the crude mixture after 15 minutes of stirring. We could speculate that this phenomenon might indicate that the reduction proceeds via Haber or Jackson mechanisms (product (**a**)), which, to date, were only associated to the catalytic hydrogenation of nitrobenzene analogues [35–37] (Figure 3).

**Scheme 3:**
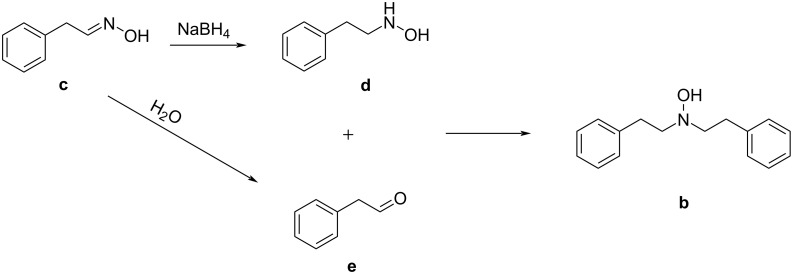
Proposed mechanism for the formation of the hydroxylamine side product **b**. *N*-Phenethylhydroxylamine (**d**), originated during the reduction process, reacts with the acetaldehyde **e** resulting from the hydrolysis of the aldoxime precursor **c**.

An attempt to identify the higher molecular masses observed by MS was made, and two intermediate structures are proposed in Figure 3. Together with (**a**), *N*,*N*-diphenethylhydroxylamine (**b**) as second product is proposed. The latter may be produced from the reaction of 2-phenylacetaldehyde (**e**) and the reduced amino product **d** via reductive alkylation [38–40] (Scheme 3).

Further research is required to clarify the formation of high molecular weight structures both prior to and following the production of the target compounds.

The application of mild heating is crucial to reach full conversion of the starting materials in the times indicated in Table 1. However, conversion to the desired products is also achievable at room temperature over 18 hour stirring with minor yield loss. Increasing the heating temperature up to 110 °C does not lead to increased product yields (for more information on the optimization process, see Supporting Information File 1, page S19).

The use of diethylenetriamine (DETA) was also investigated to evaluate its impact on the extraction process and copper(II) removal. However, the addition of DETA led to decreased yields and a deterioration of the phase separation. It was observed that using a 20% aqueous sodium hydroxide solution, instead of 35%, negatively impacted phase separation, making the extraction process more time-consuming. Additionally, the effect of the addition order of the reagents was evaluated, and the results are also provided in Supporting Information File 1.

Furthermore, methanol, 2-propanol, and water were independently tested as reaction solvents. Solubility issues, which lead to the formation of dense suspensions and precipitation of the starting materials, make these solvents unsuitable for this reaction and the isolation of the products troublesome. The use of 2-propanol/water (2:1), together with the application of heat, ensures optimal solubility of the species involved. Moreover, the workup procedure is simplified thanks to the ability of 2-propanol to partition from the sodium hydroxide aqueous solution, which allows prompt extraction of the products.

Once 2-propanol is evaporated, the products can also be isolated as free amines by dissolving the residue in diethyl ether, decanting it into another flask, and concentrating in vacuo.

Scalability was briefly investigated, and the procedure ensures minor yield loss up to 10 mmol scale of the starting material.

## Conclusion

In summary, the presented procedure represents a simple, higher-yielding, and faster alternative to the conventional reductive methods used to date for the synthesis of substituted phenethylamines from their α,β-unsaturated nitroalkene analogues. Furthermore, the NaBH_4_/CuCl_2_ system is effective at reducing nitro and ester functionalities on aromatic structures, while leaving intact benzoic acid, amido- and halogenated aromatic compounds.

## Experimental

NMR spectra were recorded on Bruker Avance 400 MHz or Bruker Avance III HD 600 MHz spectrometers. Residual solvent peaks (CDCl_3_, D_2_O, CD_3_OD, (CD_3_)_2_SO) were used as internal standard (7.26, 4.79, 3.31, and 2.50 ppm for ^1^H, and 77.16, 49, and 39.52 ppm for ^13^C, respectively). UPLC-MS analyses were performed on a Waters Acquity H-class UPLC with a Sample Manager FTN and a TUV dual wavelength detector coupled to a QDa single quadrupole analyzer using electrospray ionization (ESI). UPLC separation was achieved with a C18 reversed-phase column (Acquity UPLC BEH C18, 2.1 mm × 50 mm, 1.7 µm) operated at 40 °C, using a linear gradient of the binary solvent system of buffer A (Milli-Q H_2_O/MeCN/formic acid, 95:5:0.1 v/v) to buffer B (MeCN/formic acid, 100:0.1 v/v) from 0 to 100% B in 3.5 min, then 1 min at 100% B, flow rate: 0.8 mL/min. Data acquisition was controlled by MassLynx ver. 4.1 and data analysis was done using Waters OpenLynx browser ver. 4.1.

Solvents were commercial HPLC grade and used without further purification. The substrates **2a**, **7a**, and **8a** were commercially available and used without further purification. The substituted β-nitrostyrenes **1a** and **3a**–**6a** were prepared as described in the literature [41]. **9a** was prepared by modification of the literature [42].

### General procedure

The desired substrate (**1a**–**9a**) (2 mmol, 1 equiv) was added in small portions to a stirring suspension of NaBH_4_ (15 mmol, 7.5 equiv) in 2-PrOH/H_2_O (8 mL, 2:1). 0.1 mL of a freshly prepared CuCl_2_ 2 M solution were added dropwise but rapidly to the vessel. The reaction was monitored by TLC and refluxed at 80 °C in either oil bath or heating mantle for the time indicated in Table 1.

**General workup procedure of the amino products (1b–8b):** Once cooled to room temperature, a 35% solution of NaOH (10 mL) was added under stirring. The mixture was extracted with 2-PrOH (3 × 10 mL), and the organic extracts were combined, thoroughly dried over MgSO_4_, and filtered.

(I): The residue was concentrated under reduced pressure and dissolved in a large amount of diethyl ether. The amino products were precipitated under stirring with an excess of 2 N HCl in diethyl ether solution and the vessel was cooled to 5 °C. The solid was filtered, washed with cold diethyl ether, and dried under reduced pressure as the amine hydrochloride salt.

(II) An excess of 4 N HCl in dioxane solution was added and the filtrate was stirred for 30 minutes. The residue was concentrated under reduced pressure, suspended in dry cold acetone, and stirred vigorously for 1 hour. The suspension was filtered and washed with a minimum amount of cold acetone to deliver the product as hydrochloride salt.

**2-Phenylethan-1-amine hydrochloride (1b):** The product was isolated by use of (II) as an amorphous white solid (83%). ^1^H NMR (600 MHz, CD_3_OD) δ 2.97 (m, *J* = 5.18 Hz, 2H), 3.18 (m, *J* = 5.24 Hz, 2H), 7.28 (m, *J* = 5.0 Hz, 3H), 7.35 (m, *J* = 7.6 Hz, 2H); ^13^C NMR (151 MHz, CD_3_OD) δ 34.55, 41.98, 128.26, 129.77, 129.99, 137.92; ESI-MS *m*/*z*: [M + 1]^+^ 121.1; found, 121.0; mp 220–221 °C.

**2-(4-Methoxyphenyl)ethan-1-amine hydrochloride (2b):** The product was isolated by use of (I) as a white solid (82%). ^1^H NMR (600 MHz, CD_3_OD) δ 2.89 (t, *J* = 7.7 Hz, 2H), 3.13 (t, *J* = 7.7 Hz, 2H), 3.78 (s, 3H), 6.91 (ddd, *J* = 8.4, 2.8, 0.2 Hz, 2H), 7.19 (ddd, *J* = 8.4, 2.5, 0.2 Hz, 2H); ^13^C NMR (151 MHz, CD_3_OD) δ 33.75, 42.14, 55.71, 115.42, 129.60, 130.78, 160.47; ESI-MS *m*/*z*: [M + 1]^+^ 151.1; found, 152.1; mp 214–216 °C.

**1-(2,5-Dimethoxyphenyl)propan-2-amine hydrochloride (3b):** The product was isolated by use of (II) as a white solid (62%). ^1^H NMR (600 MHz, CD_3_OD) δ 1.26 (d, *J* = 6.60 Hz, 3H), 2.82 (m, *J* = 6.92 Hz, 1H), 2.95 (m, *J* = 6.60 Hz, 1H), 3.56 (m, *J* = 6.51 Hz, 1H), 3.75 (s, 3H), 3.81 (s, 3H), 6.79 (m, *J* = 2.94 Hz, 1H), 6.84 (dd, *J* = 2.43, 8.85 Hz, 1H), 6.93 (m, *J* = 8.94 Hz, 1H); ^13^C NMR (151 MHz, CD_3_OD) δ 18.56, 36.85, 49.22, 56.12, 56.24, 112.81, 114.06, 118.63, 126.24, 153.17, 155.14; ESI-MS *m*/*z*: [M + 1]^+^ 135.1; found, 136.2; mp 115–117 °C.

**2-(2,5-Dimethoxyphenyl)ethan-1-amine hydrochloride (4b):** The product was isolated by use of (I) as a white solid (82%). ^1^H NMR (600 MHz, (CD_3_)_2_SO) δ 2.81 (t, *J* = 7.8 Hz, 2H), 2.97 (t, *J* = 7.8 Hz, 2H), 3.70 (s, 3H), 3.75 (s, 3H), 6.78 (m, *J* = 3.1 Hz, 1H), 6.81 (dd, *J* = 3.09, 8.82 Hz, 1H), 6.92 (m, *J* = 8.9 Hz, 1H); ^13^C NMR (151 MHz, (CD_3_)_2_SO) δ 28.14, 38.65, 55.32, 55.79, 111.78, 112.18, 116.45, 126.03, 151.25, 153.05; ESI-MS *m*/*z*: [M + 1]^+^ 181.1; found, 182.2; mp 138–140 °C.

**2-(2,5-Dimethoxy-4-methylphenyl)ethan-1-amine hydrochloride (5b):** The product was isolated by use of (II) as a white solid (65%). ^1^H NMR (600 MHz, CD_3_OD) δ 2.18 (s, 3H), 2.92 (t, *J* = 7.38 Hz, 2H), 3.12 (t, *J* = 7.38 Hz, 2H), 3.78 (s, 3H), 3.80 (s, 3H), 6.76 (s, 1H), 6.81 (s, 1H); ^13^C NMR (151 MHz, CD_3_OD) δ 16.27, 29.81, 41.07, 56.33, 56.48, 114.24, 114.96, 123.38, 127.73, 152.65, 153.22; ESI-MS *m*/*z*: [M + 1]^+^ 195.1; found, 196.2; mp 213–215 °C.

**2-(2,5-Dimethoxy-4-(trifluoromethyl)phenyl)ethan-1-amine hydrochloride (6b):** The product was isolated by use of (II) as a white solid (71%). ^1^H NMR (400 MHz, CD_3_OD) δ 3.03 (t, *J* = 7.38 Hz, 2H), 3.18 (m, *J* = 3.76 Hz, 2H), 3.87 (s, 3H), 3.88 (s, 3H), 7.10 (s, 1H), 7.16 (s, 1H); ^13^C NMR (151 MHz, CD_3_OD) δ 29.96, 40.85, 56.11, 56.26, 112.74, 113.86, 113.87, 117.99, 126.84, 153.15, 155.24; ESI-MS *m*/*z*: [M + 1]^+^ 249.1; found, 250.1; mp 260–261 °C.

**Aniline hydrochloride (7b):** The product formation was monitored by TLC using Hex/EtOAc/TEA (3:7:0.1). The product was isolated by use of (I) as a white solid (96%). ^1^H NMR (600 MHz, D_2_O) δ 7.40 (m, *J* = 2.96 Hz, 2H), 7.51 (m, *J* = 1.66 Hz, 1H), 7.56 (m, *J* = 1.79 Hz, 2H); ^13^C NMR (151 MHz, D_2_O) δ 109.59, 122.50, 128.67, 130.07; ESI-MS *m*/*z*: [M + 1]^+^ 93.1; found, 94,2; mp 196–197 °C.

***p*-Bromoaniline hydrochloride (8b):** The product formation was monitored by TLC using pure pentane. The product was isolated by use of (I) as a bright white powder (97%). ^1^H NMR (600 MHz, D_2_O) δ 7.31 (ddd, *J* = 8.5, 2.6, 0.3 Hz, 2H), 7.66 (ddd, *J* = 8.5, 2.6, 0.3 Hz, 2H); ^13^C NMR (151 MHz, D_2_O) δ 122.41, 124.77, 129.01, 133.06; ESI-MS *m*/*z*: [M + 1]^+^ 171.0; found, 171.1; mp 190–191 °C.

**Benzyl alcohol (9b):** The product formation was monitored by TLC using Hex/EtOAc (6:1). Once cooled to room temperature, the mixture was acidified with 20% HCl solution and extracted with DCM (3 × 15 mL). The organic extracts were combined, dried over MgSO_4_, and concentrated under reduced pressure to deliver **9b** as colorless liquid (92%). ^1^H NMR (400 MHz, CDCl_3_) δ 1.87 (br, 1H), 4.69 (s, 2H), 7.31 (m, *J* = 2.67 Hz, 1H), 7.37 (m, *J* = 2.30 Hz, 4H); ^13^C NMR (151 MHz, CDCl_3_) δ 65.48, 127.12, 127.79, 128.69, 140.97; ESI-MS *m*/*z*: [M + 1]^+^ 108.1; found, 109.1.

## Supporting Information

File 1 ^1^H and ^13^C NMR spectra of the synthesized compounds, the optimization table, and ESI-MS spectra for the synthesis of **4b**.

## Data Availability

All data that supports the findings of this study is available in the published article and/or the supporting information of this article.
