# Supplementary material for: Facile one-pot reduction of β-nitrostyrenes to phenethylamines using sodium borohydride and copper(II) chloride
Source: Beilstein J Org Chem. 2025 Jan 7;21:39–46. doi: 10.3762/bjoc.21.4 (PMC11729678; doi:10.3762/bjoc.21.4)
Supplement: File 1 — 1H and 13C NMR spectra of the synthesized compounds, the optimization table, and ESI-MS spectra for the synthesis of 4b. [file Beilstein_J_Org_Chem-21-39-s001.pdf]

BEILSTEIN JOURNAL OF ORGANIC CHEMISTRY

Supporting Information

for

Facile one-pot reduction of β-nitrostyrenes to phenethylamines using sodium borohydride and copper(II) chloride

Laura D'Andrea and Simon Jademyr

Beilstein J. Org. Chem. **2025,** *21,* 39–46. doi:10.3762/bjoc.21.4

^{1}H and ^{13}C NMR spectra of the synthesized compounds, the optimization table, and ESI-MS spectra for the synthesis of 4b

License and Terms: This is a supporting information file under the terms of the Creative Commons Attribution License (https://creativecommons.org/licenses/by/4.0). Please note that the reuse, redistribution and reproduction in particular requires that the author(s) and source are credited and that individual graphics may be subject to special legal provisions.
The license is subject to the *Beilstein Journal of Organic Chemistry* terms and conditions: (https://www.beilstein-journals.org/bjoc/terms)

Table of Contents

2-Phenylethan-1-amine hydrochloride (1b) S**1**
^{1}H NMR spectrum S1
^{13}C NMR spectrum S2
2-(4-Methoxyphenyl)ethan-1-amine hydrochloride (2b) S**3**
^{1}H NMR spectrum S3
^{13}C NMR spectrum S4
1-(2,5-Dimethoxyphenyl)propan-2-amine hydrochloride (3b) S**5**
^{1}H NMR spectrum S5
^{13}C NMR spectrum S6
2-(2,5-Dimethoxyphenyl)ethan-1-amine hydrochloride (4b) S**7**
^{1}H NMR spectrum S7
^{13}C NMR spectrum S8
2-(2,5-Dimethoxy-4-methylphenyl)ethan-1-amine hydrochloride (5b) S**9**
^{1}H NMR spectrum S9
^{13}C NMR spectrum S10
2-(2,5-Dimethoxy-4-(trifluoromethyl)phenyl)ethan-1-amine hydrochloride (6b) S**11**
^{1}H NMR spectrum S11
^{13}C NMR spectrum S12
Aniline hydrochloride (7b) S**13**
^{1}H NMR spectrum S13
^{13}C NMR spectrum S14
***p*-Bromoaniline hydrochloride (8b)** S**15**
^{1}H NMR spectrum S15
^{13}C NMR spectrum S16
Benzyl alcohol (9b) S**17**
^{1}H NMR spectrum S17
^{13}C NMR spectrum S18
Optimization table S**19**
ESI/MS spectra of 4b S**20-22**
References S**23**

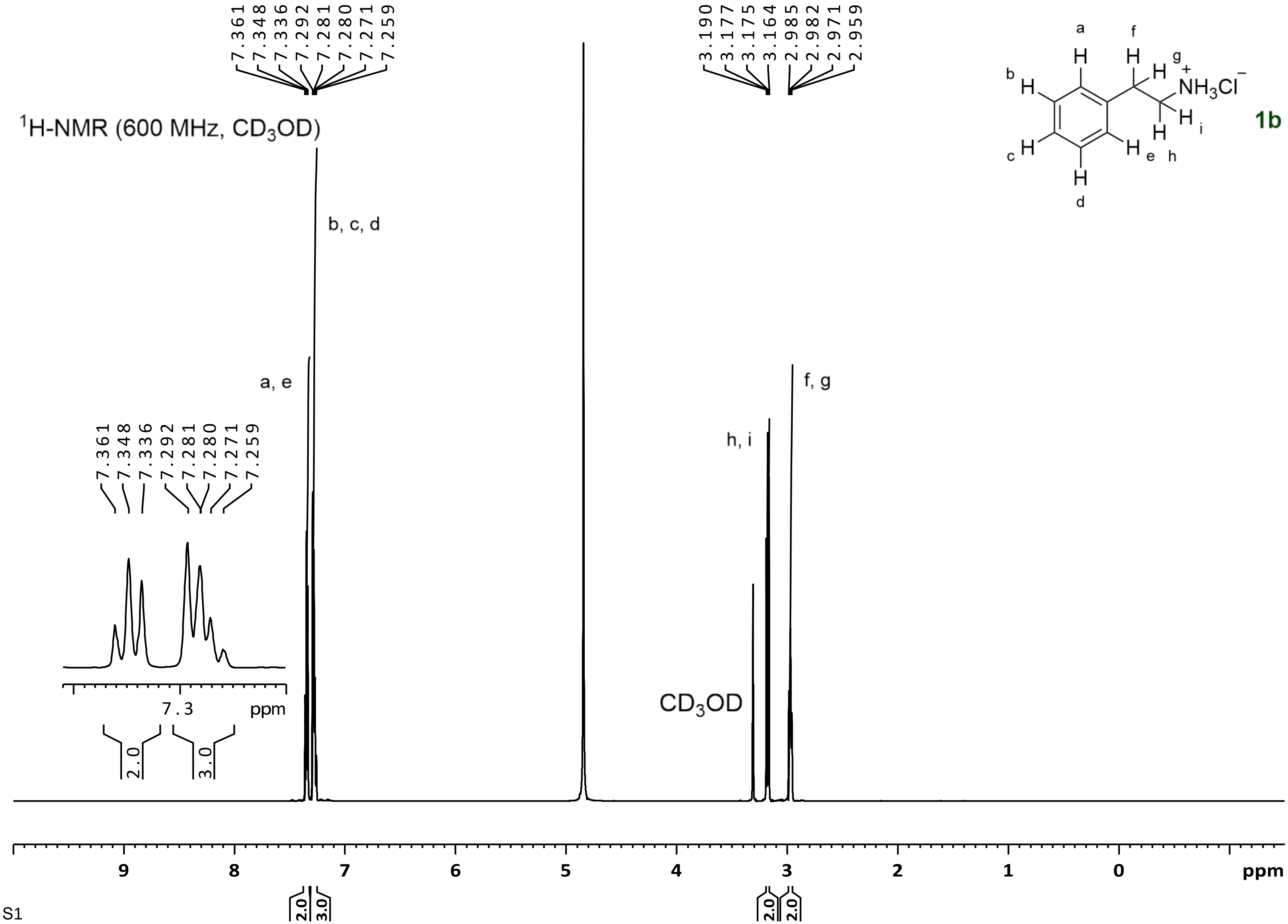
1H-NMR (600 MHz, CD3OD)
7.361 7.348 7.336 7.292 7.281 7.280 7.271 7.259
3.190 3.177 3.175 3.164 2.985 2.982 2.971 2.959
b, c, d
a, e
h, i
f, g
CD3OD
1b
a b c d e f g h i
NH3Cl
7.3 ppm
2.0
3.0
9 8 7 6 5 4 3 2 1 0 ppm
2.0 3.0 2.0 2.0

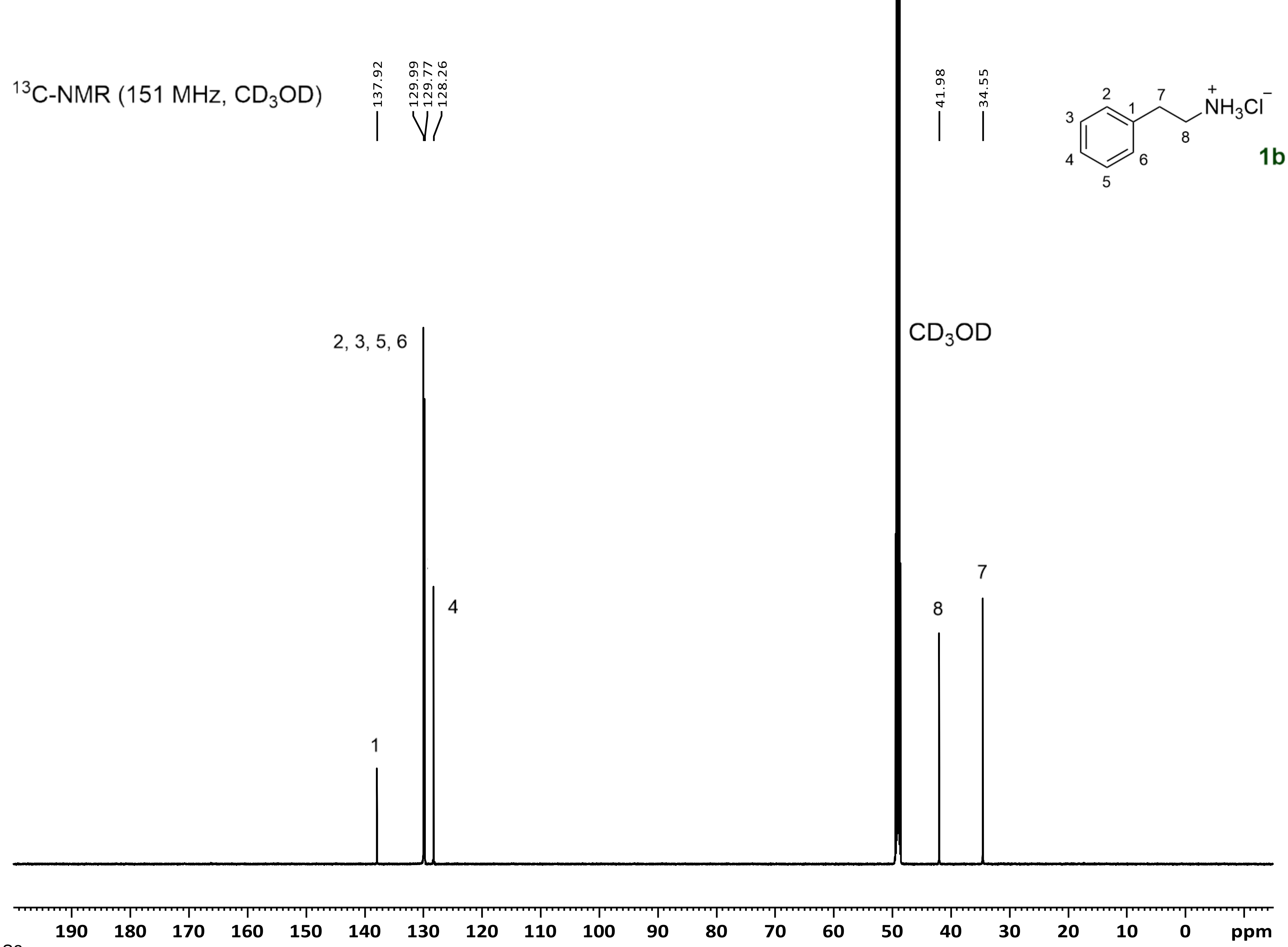

^{13}C-NMR (151 MHz, CD_3OD)
137.92
129.99
129.77
128.26
41.98
34.55
2, 3, 5, 6
4
1
CD_3OD
8
7
$\overset{+}{N}H_3Cl^-$
1b
190 180 170 160 150 140 130 120 110 100 90 80 70 60 50 40 30 20 10 0 ppm

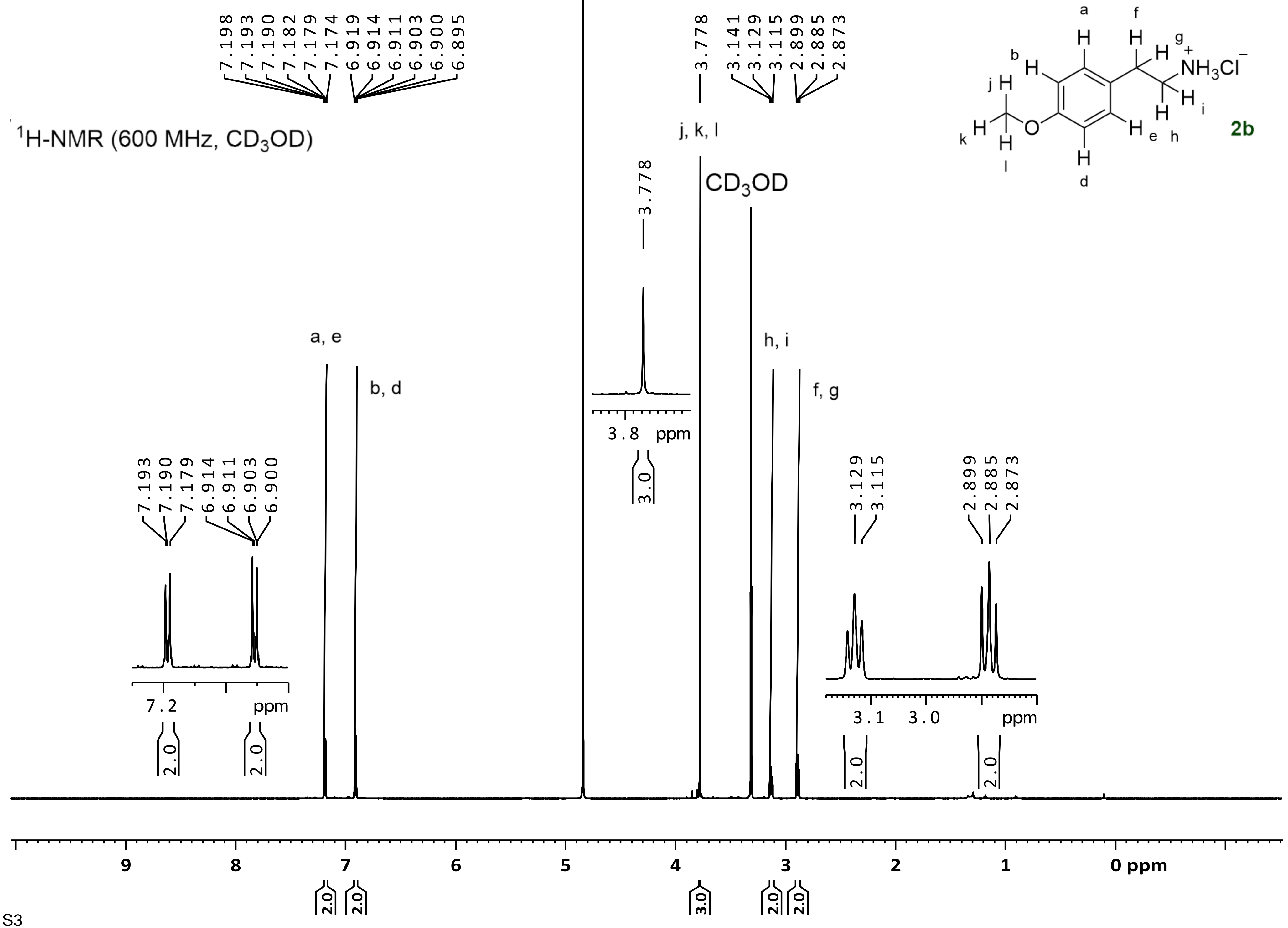

7.198 7.193 7.190 7.182 7.179 7.174 6.919 6.914 6.911 6.903 6.900 6.895
3.778
3.141 3.129 3.115 2.899 2.885 2.873
^{1}H-NMR (600 MHz, CD3OD)
2b
a, e
b, d
j, k, l
CD3OD
h, i
f, g
3.778
3.8 ppm
3.0
7.193 7.190 7.179 6.914 6.911 6.903 6.900
7.2 ppm
2.0
2.0
3.129 3.115
2.899 2.885 2.873
3.1 3.0 ppm
2.0
2.0
9 8 7 6 5 4 3 2 1 0 ppm
2.0 2.0 3.0 2.0 2.0

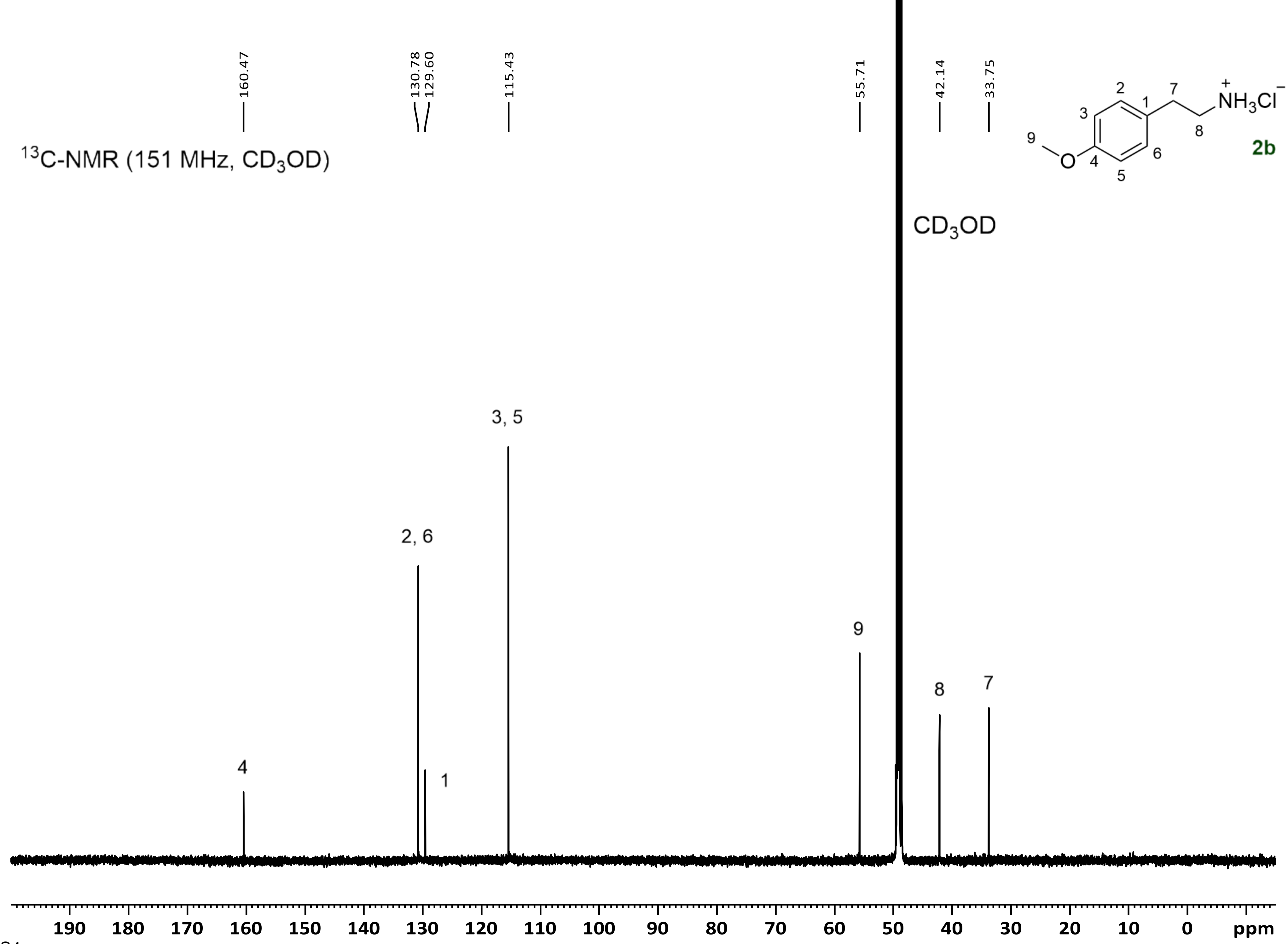

160.47
130.78
129.60
115.43
55.71
42.14
33.75
^{13}C-NMR (151 MHz, CD_3OD)
CD_3OD
2b
4
2, 6
1
3, 5
9
8
7
190 180 170 160 150 140 130 120 110 100 90 80 70 60 50 40 30 20 10 0 ppm

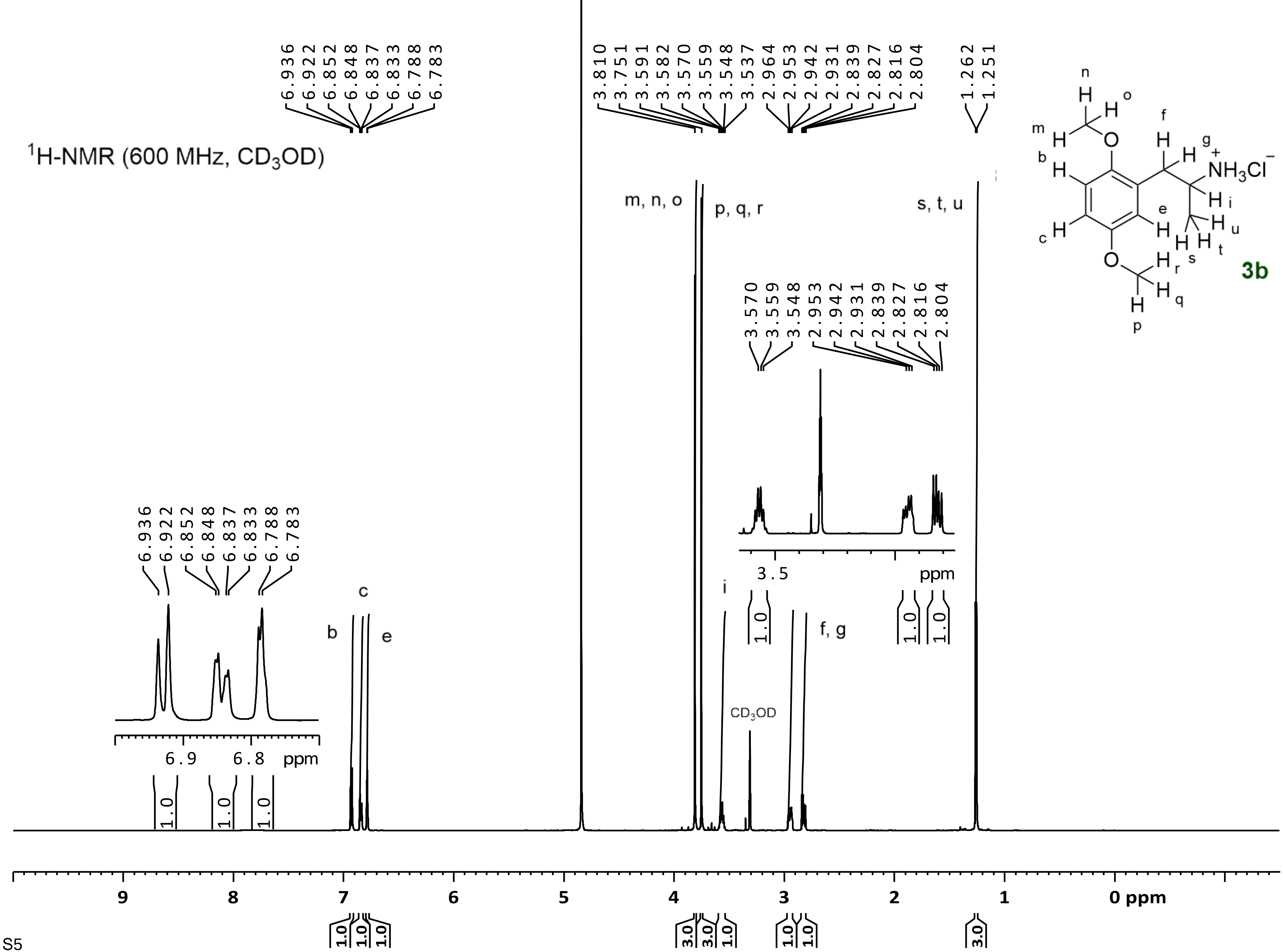

1H-NMR (600 MHz, CD3OD)
6.936 6.922 6.852 6.848 6.837 6.833 6.788 6.783
3.810 3.751 3.591 3.582 3.570 3.559 3.548 3.537 2.964 2.953 2.942 2.931 2.839 2.827 2.816 2.804
1.262 1.251
m, n, o
p, q, r
s, t, u
b
c
e
i
f, g
CD3OD
3b
9 8 7 6 5 4 3 2 1 0 ppm
1.0 1.0 1.0 3.0 3.0 1.0 1.0 1.0 3.0

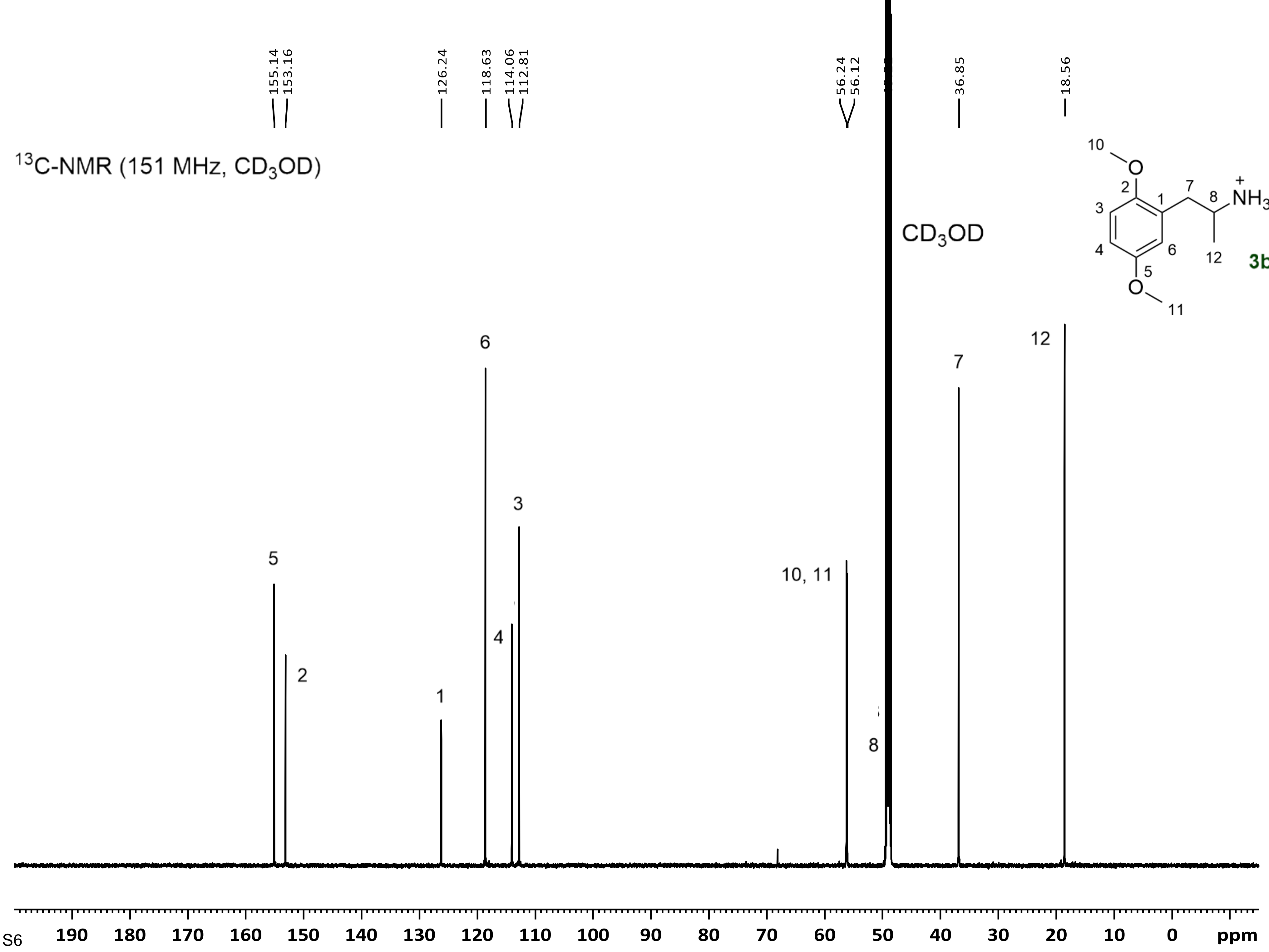

155.14
153.16
126.24
118.63
114.06
112.81
56.24
56.12
36.85
18.56
^{13}C-NMR (151 MHz, CD_3OD)
CD_3OD
3b
5
2
1
6
4
3
10, 11
8
7
12
190 180 170 160 150 140 130 120 110 100 90 80 70 60 50 40 30 20 10 0 ppm

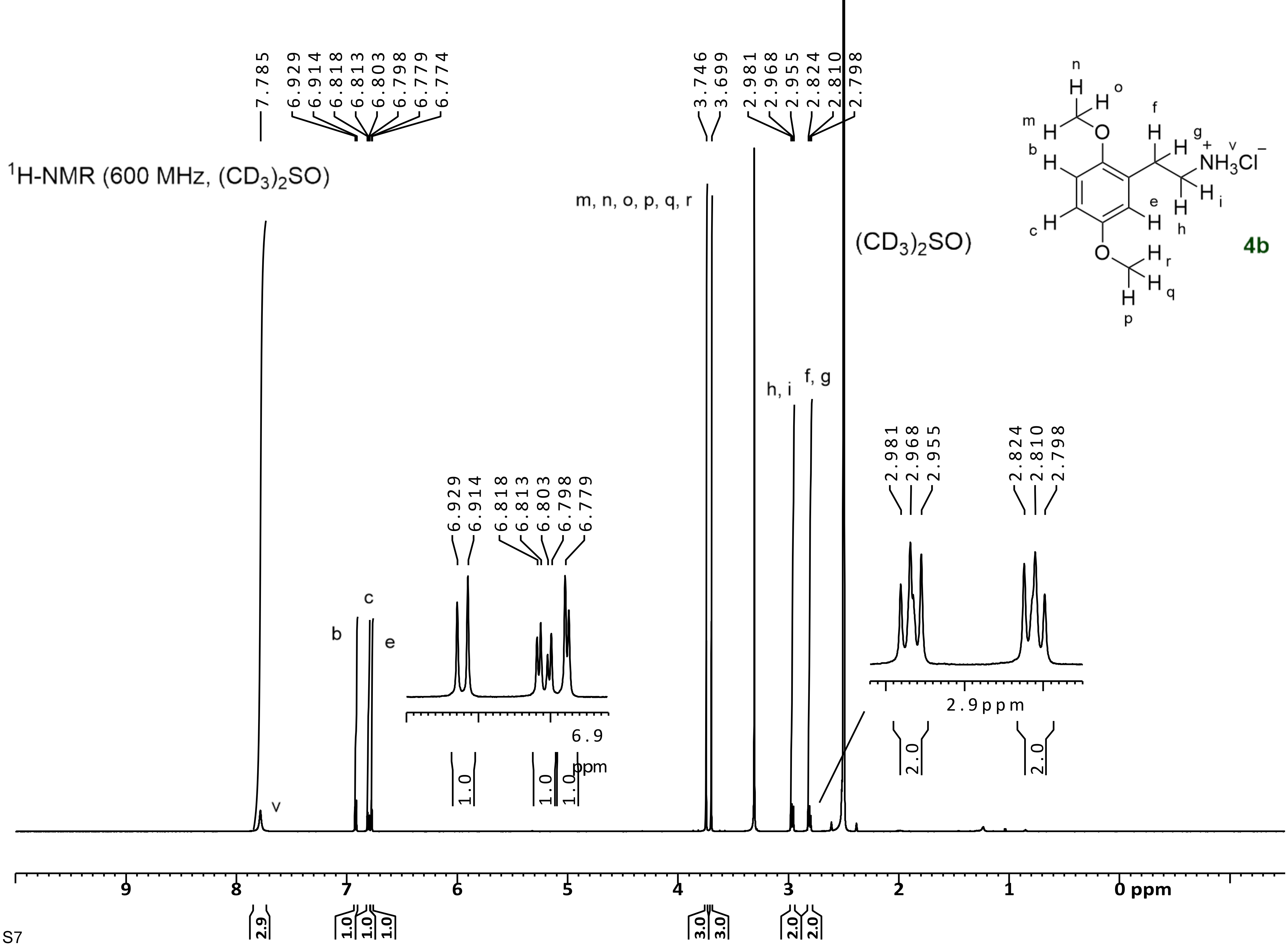

^{1}H-NMR (600 MHz, $(CD_3)_2SO$)
7.785
6.929
6.914
6.818
6.813
6.803
6.798
6.779
6.774
3.746
3.699
2.981
2.968
2.955
2.824
2.810
2.798
m, n, o, p, q, r
h, i
f, g
$(CD_3)_2SO$
b
c
e
v
4b
$NH_3^+ Cl^-$
6.9 ppm
2.9 ppm
1.0
2.0
2.9
3.0
9
8
7
6
5
4
3
2
1
0 ppm

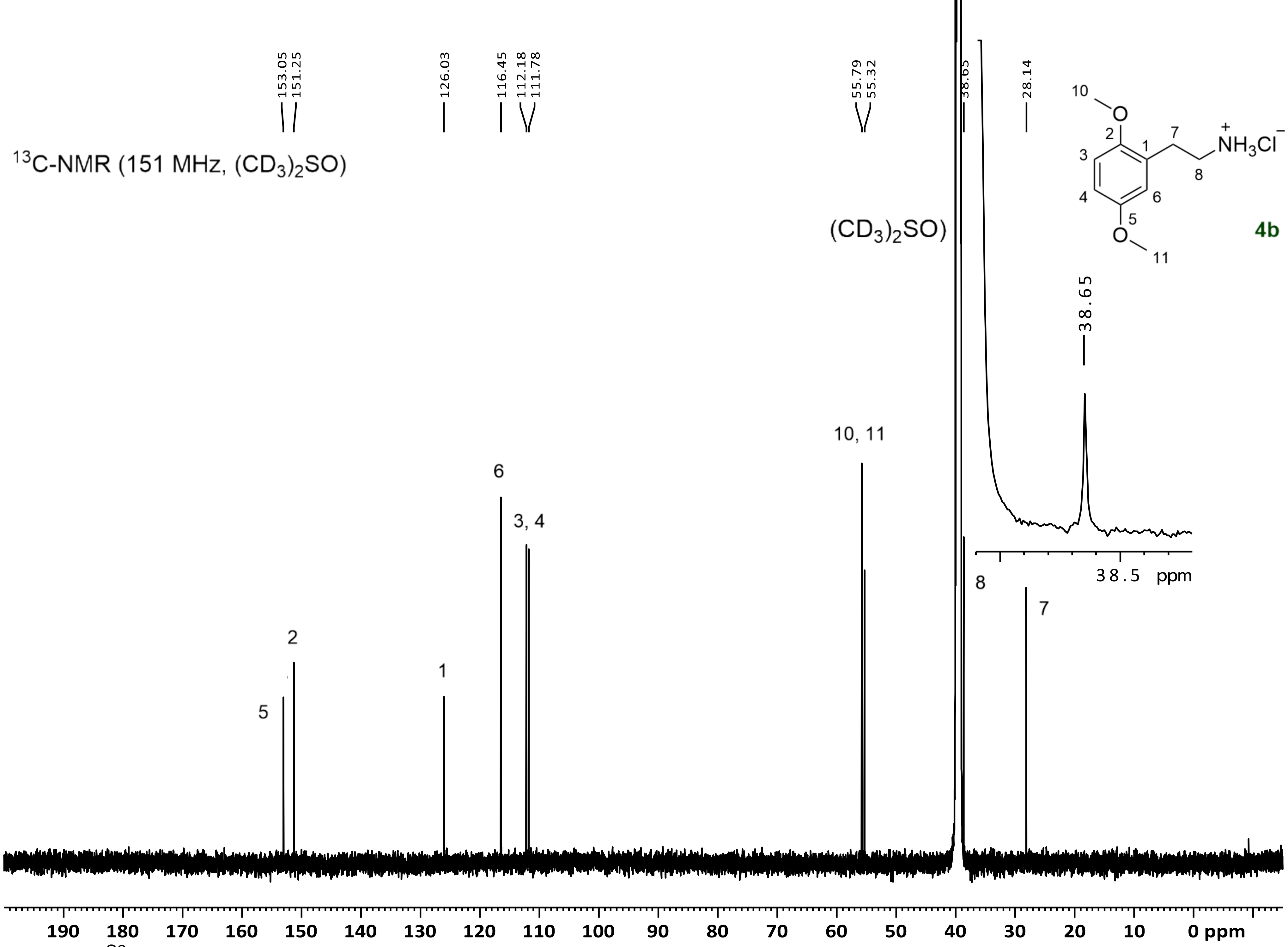

153.05
151.25
126.03
116.45
112.18
111.78
55.79
55.32
38.65
28.14
^{13}C-NMR (151 MHz, $(CD_3)_2SO$)
$(CD_3)_2SO$
4b
10, 11
6
3, 4
2
1
5
8
7
38.5 ppm
190 180 170 160 150 140 130 120 110 100 90 80 70 60 50 40 30 20 10 0 ppm

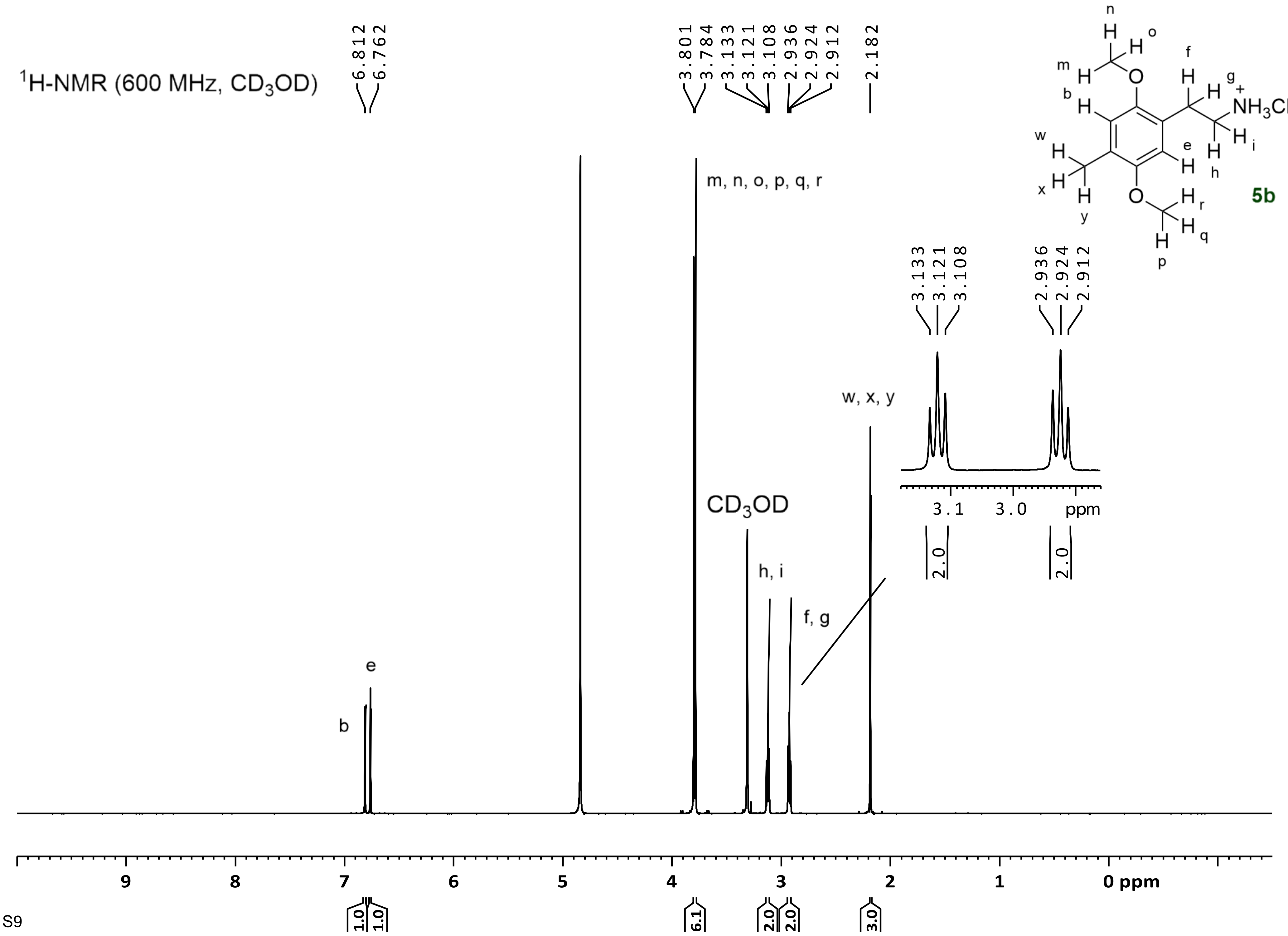

^{1}H-NMR (600 MHz, CD_3OD)
6.812
6.762
3.801
3.784
3.133
3.121
3.108
2.936
2.924
2.912
2.182
m, n, o, p, q, r
w, x, y
CD_3OD
h, i
f, g
e
b
5b
NH_3Cl^-
ppm
9
8
7
6
5
4
3
2
1
0 ppm
1.0
1.0
6.1
2.0
2.0
3.0

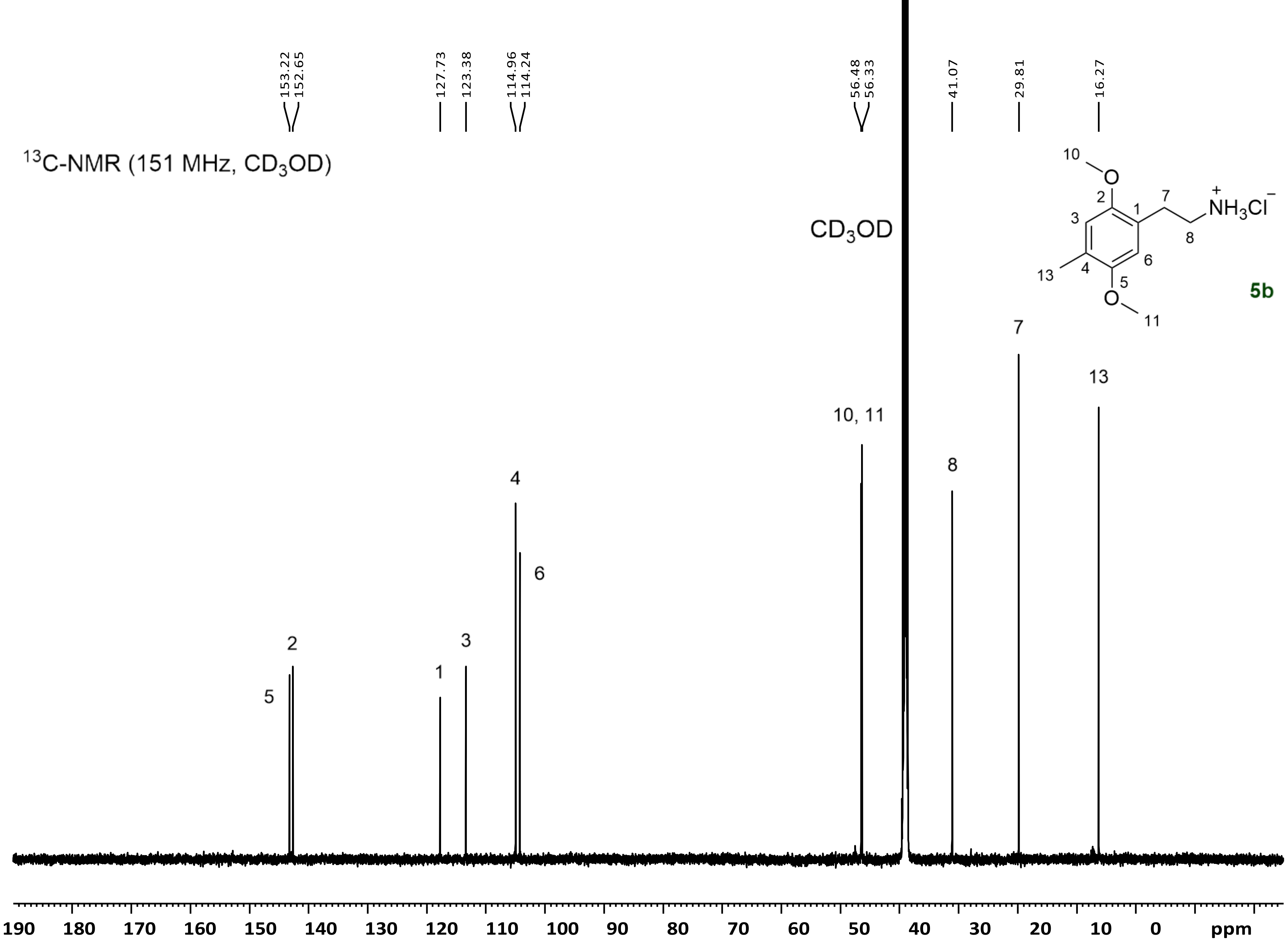

153.22
152.65
127.73
123.38
114.96
114.24
56.48
56.33
41.07
29.81
16.27
13C-NMR (151 MHz, CD3OD)
CD3OD
5b
10, 11
7
13
8
4
6
2
5
1
3
190 180 170 160 150 140 130 120 110 100 90 80 70 60 50 40 30 20 10 0 ppm

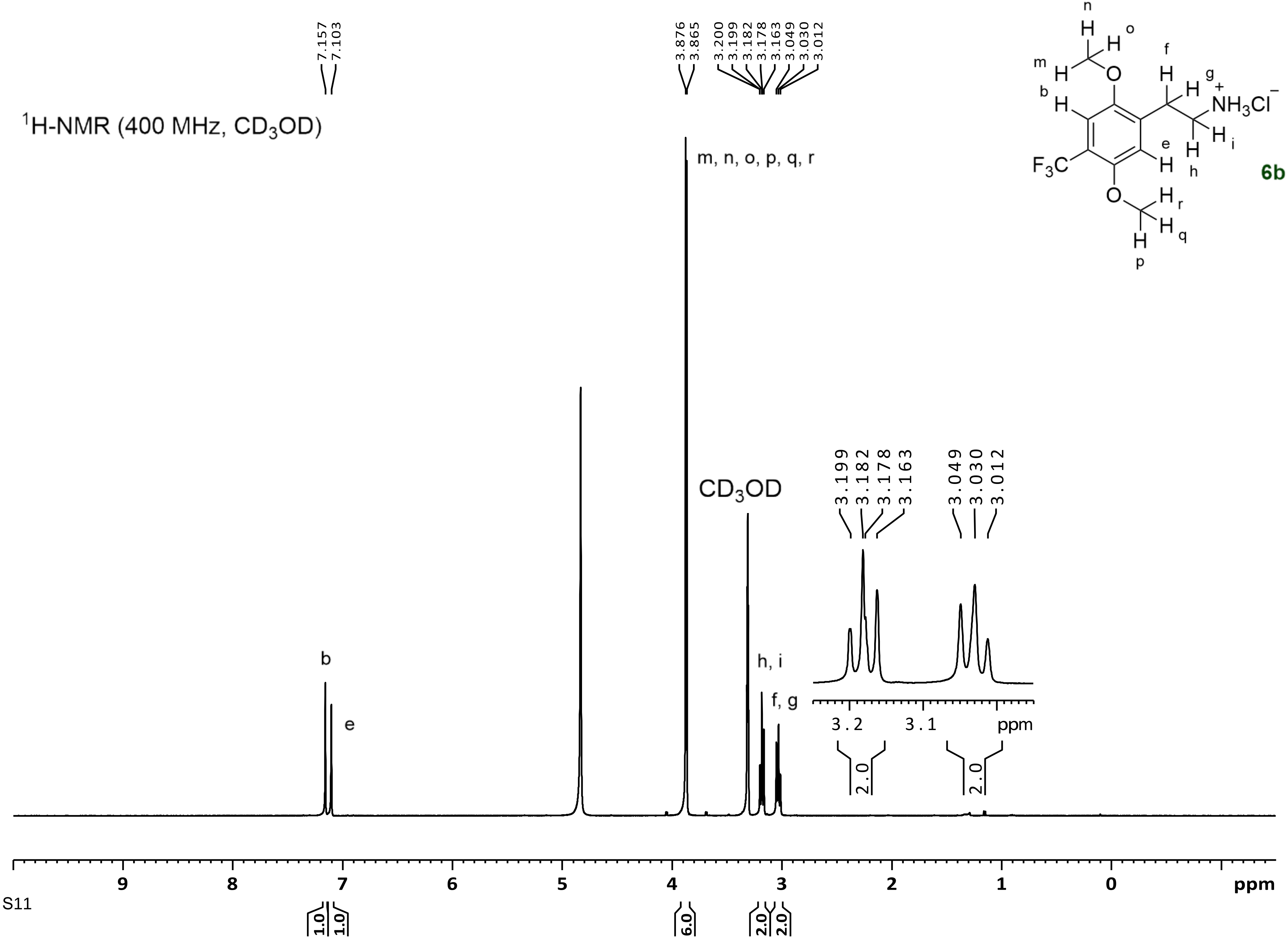

1H-NMR (400 MHz, CD3OD)
7.157
7.103
3.876
3.865
3.200
3.199
3.182
3.178
3.163
3.049
3.030
3.012
m, n, o, p, q, r
CD3OD
h, i
f, g
b
e
6b
NH3Cl
F3C
ppm
9 8 7 6 5 4 3 2 1 0
1.0 1.0 6.0 2.0 2.0

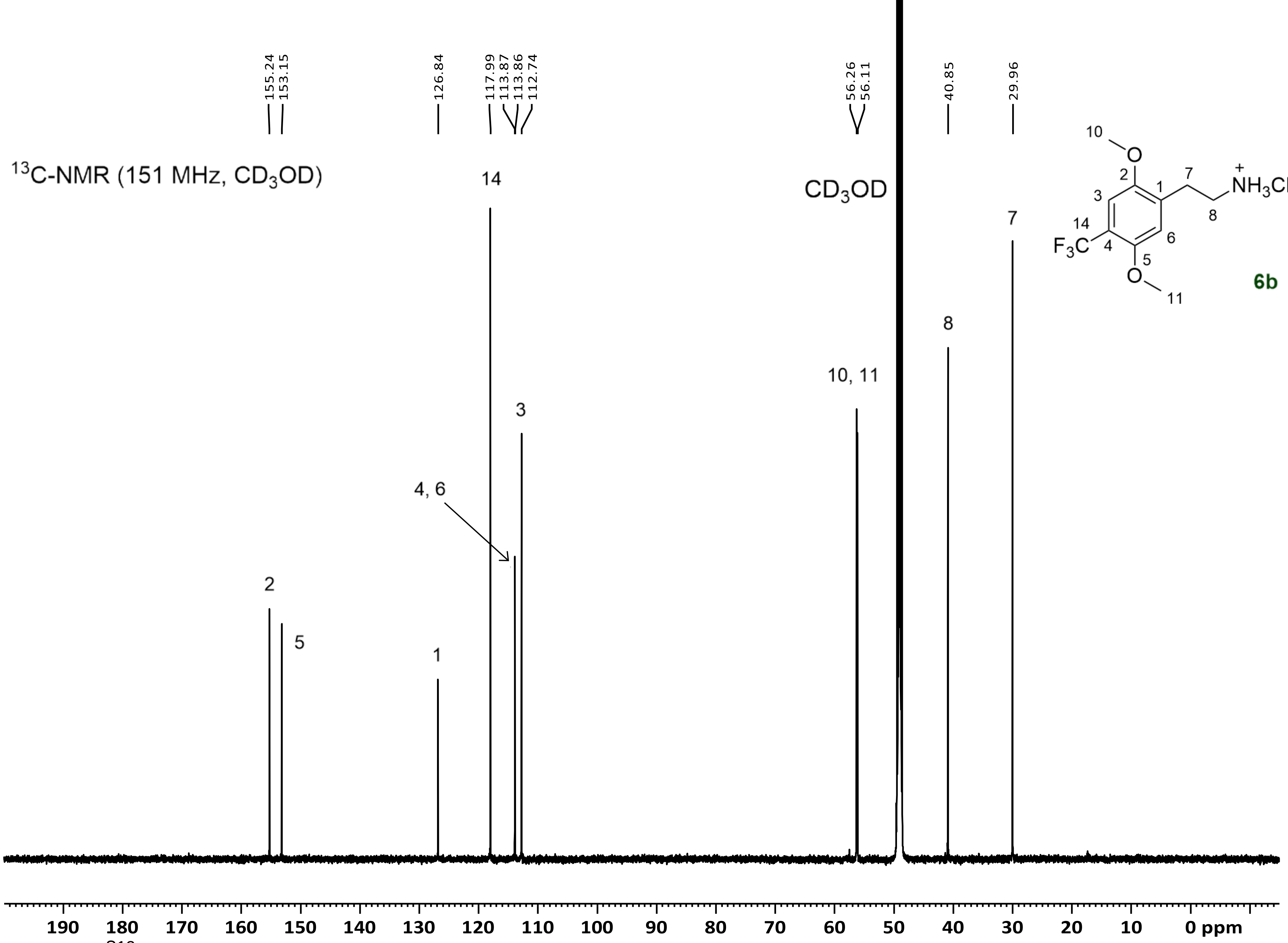
155.24
153.15
126.84
117.99
113.87
113.86
112.74
56.26
56.11
40.85
29.96
^{13}C-NMR (151 MHz, CD_3OD)
14
CD_3OD
7
8
10, 11
3
4, 6
2
5
1
10
2
7
1
3
14
4
6
5
11
8
F_3C
$\overset{+}{N}H_3Cl^-$
O
O
6b
190
180
170
160
150
140
130
120
110
100
90
80
70
60
50
40
30
20
10
0 ppm

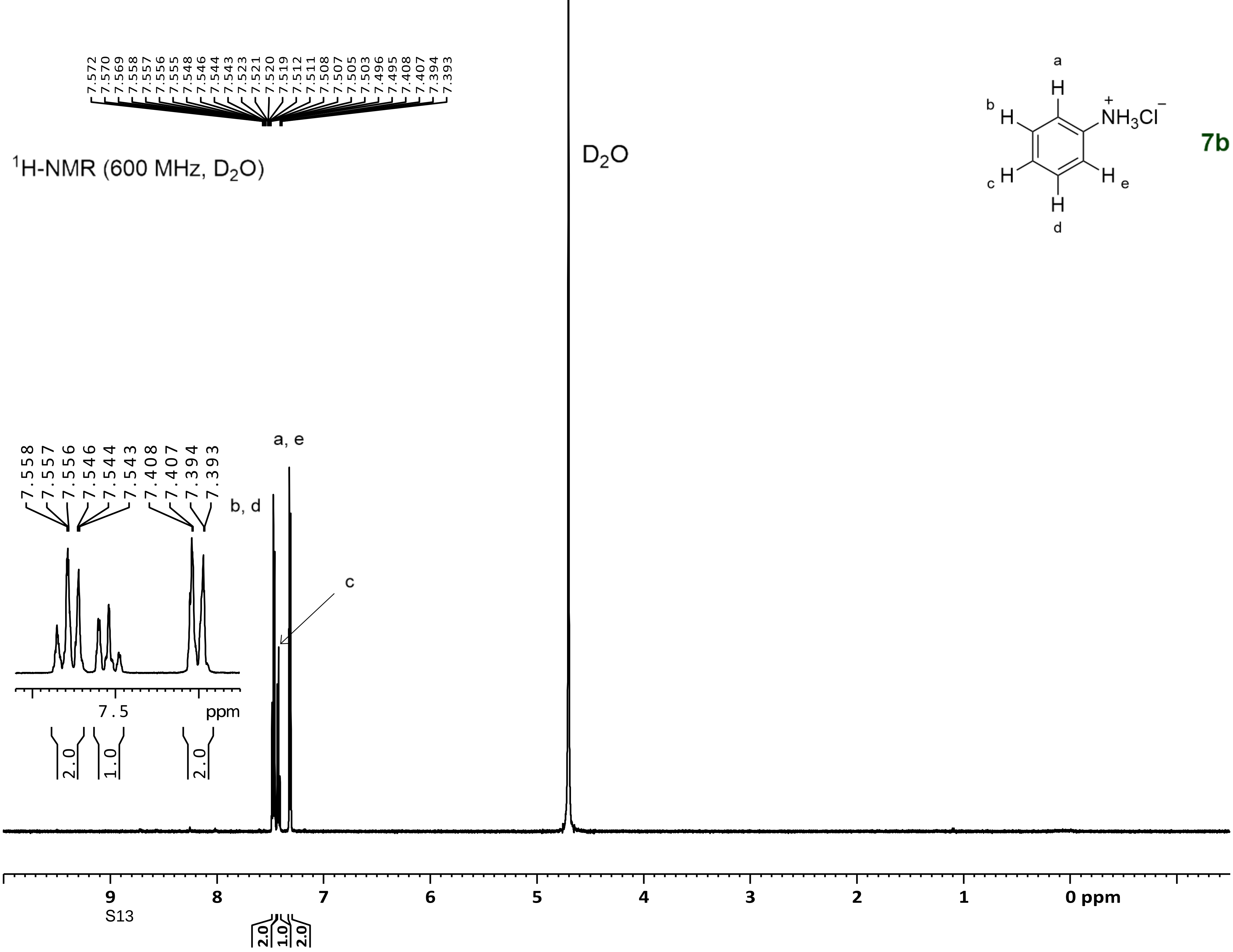
7.572
7.570
7.569
7.558
7.557
7.556
7.555
7.548
7.546
7.544
7.543
7.523
7.521
7.520
7.519
7.512
7.511
7.508
7.507
7.505
7.503
7.496
7.495
7.408
7.407
7.394
7.393
^{1}H-NMR (600 MHz, D_2O)
D_2O
a
b
c
d
e
$\overset{+}{N}H_3Cl^-$
7b
a, e
b, d
c
7.558
7.557
7.556
7.546
7.544
7.543
7.408
7.407
7.394
7.393
7.5
ppm
2.0
1.0
2.0
9
8
7
6
5
4
3
2
1
0 ppm
2.0
1.0
2.0

^{13}C-NMR (151 MHz, D_2O)

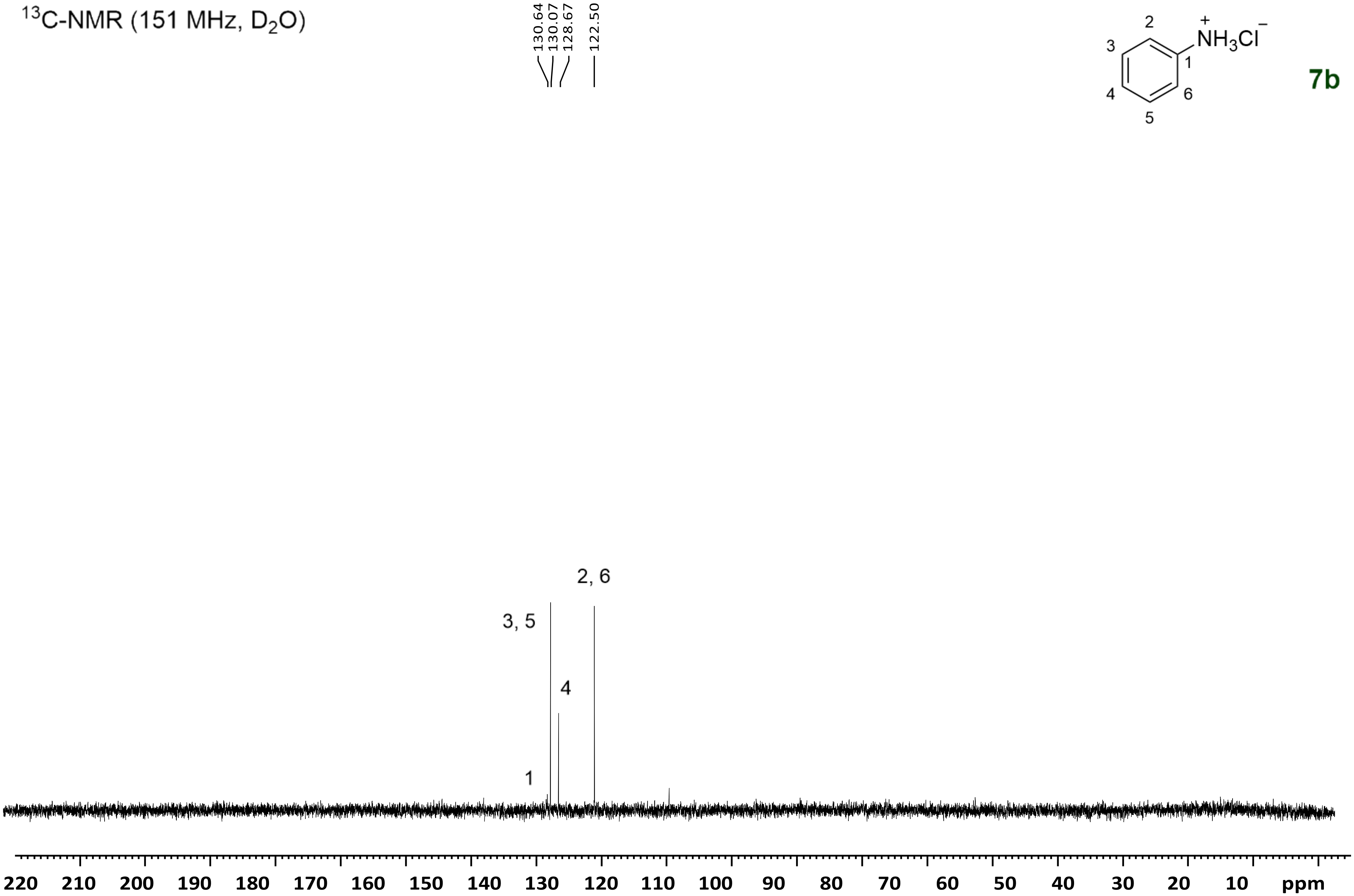

7b

^{1}H-NMR (600 MHz, D_2O)

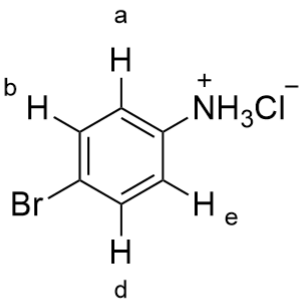

8b

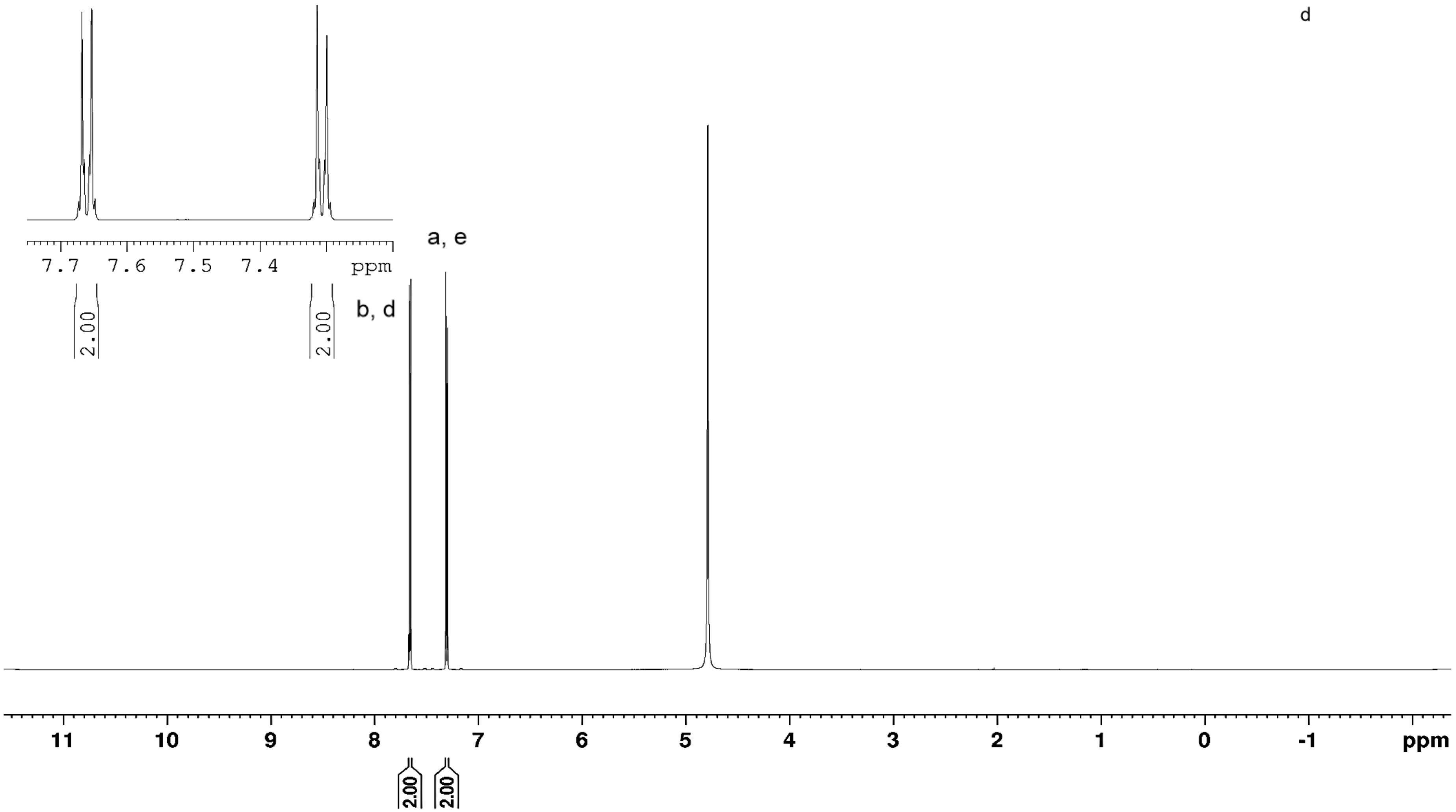

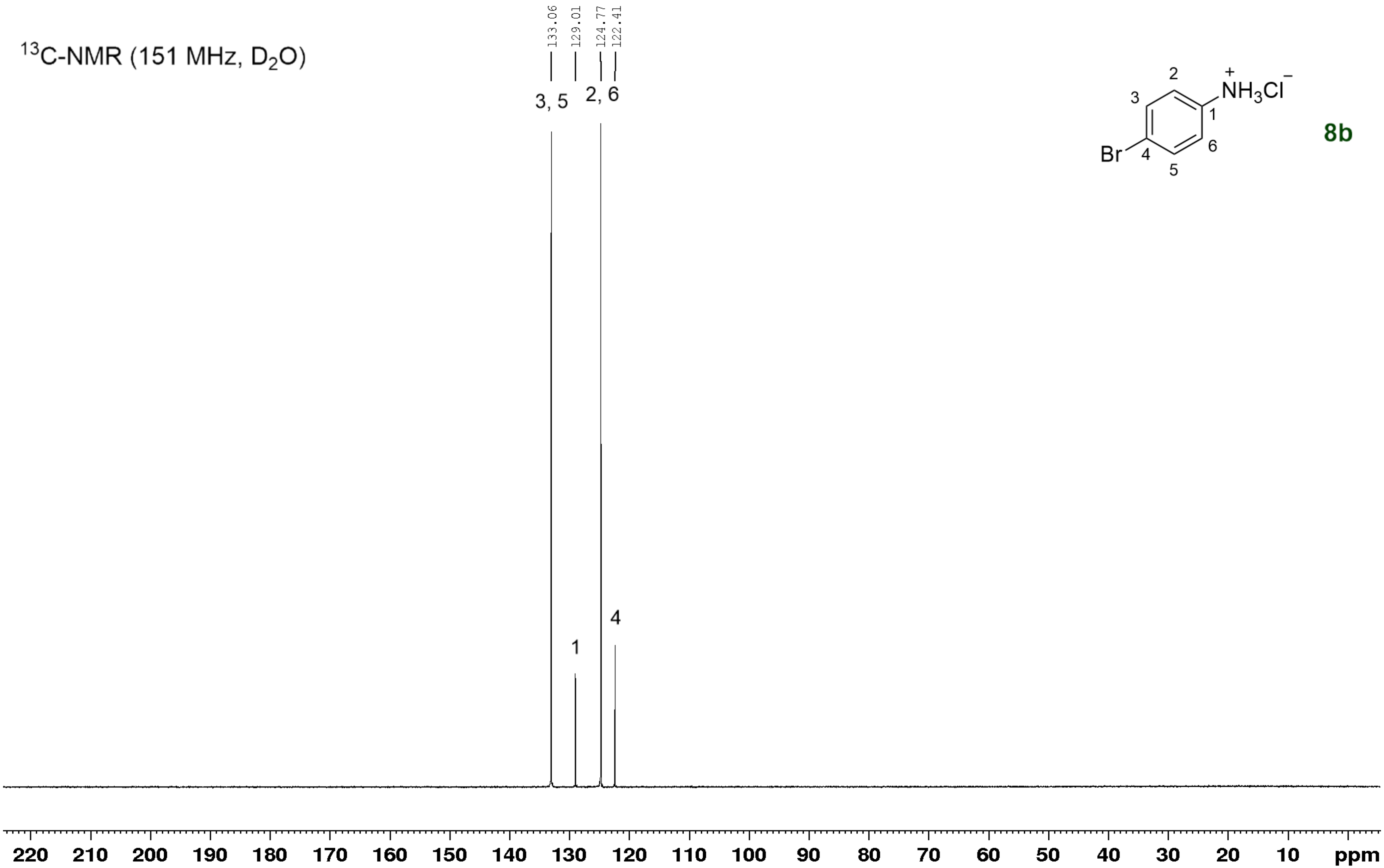

^{13}C-NMR (151 MHz, D_2O)
133.06
129.01
124.77
122.41
3, 5
2, 6
1
4
8b
Br
NH3Cl
220 210 200 190 180 170 160 150 140 130 120 110 100 90 80 70 60 50 40 30 20 10 ppm

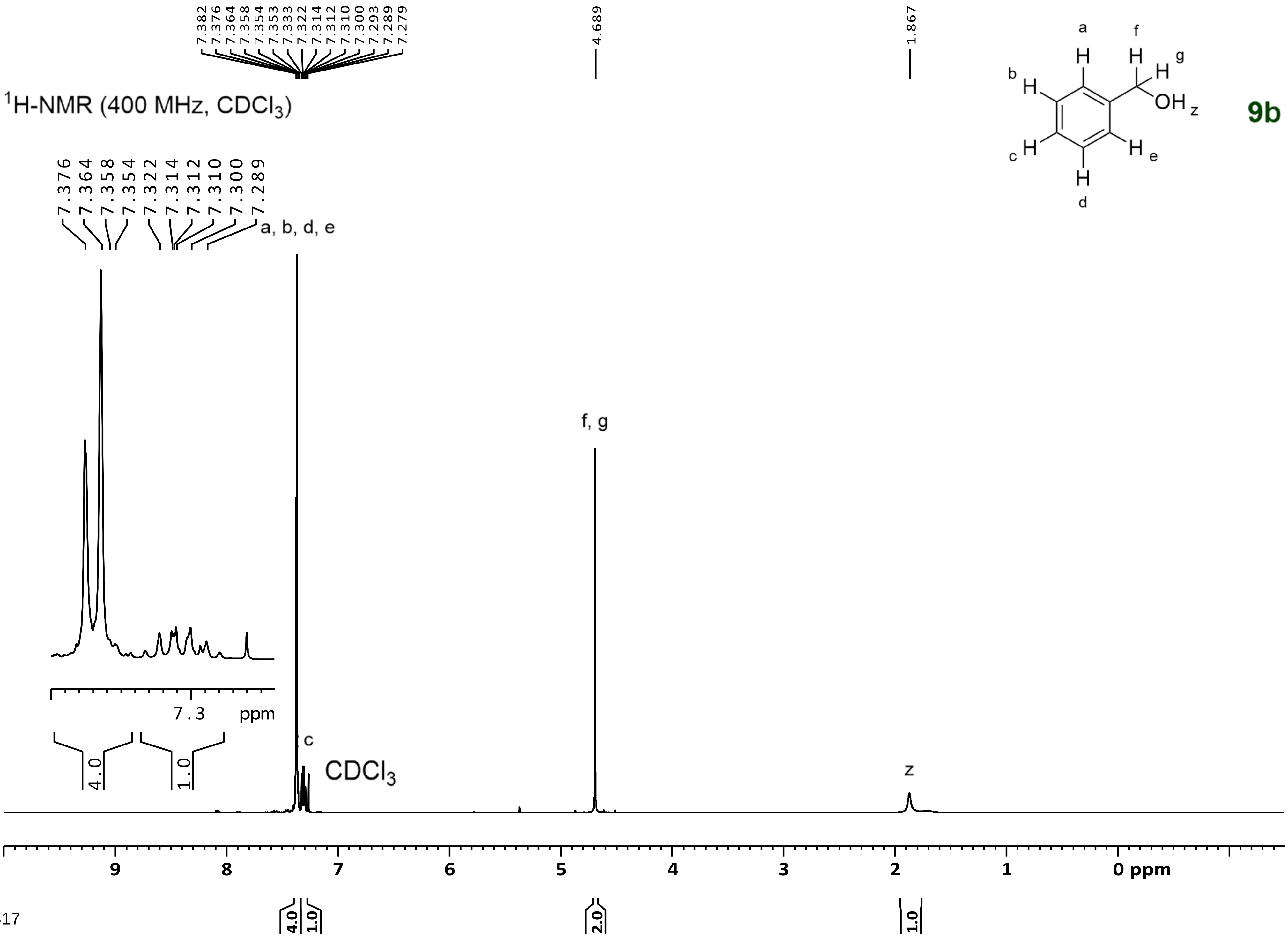

7.382 7.376 7.364 7.358 7.354 7.353 7.333 7.322 7.314 7.312 7.310 7.300 7.293 7.289 7.279
4.689
1.867
1H-NMR (400 MHz, CDCl3)
7.376 7.364 7.358 7.354 7.322 7.314 7.312 7.310 7.300 7.289
a, b, d, e
f, g
c
CDCl3
z
7.3 ppm
4.0
1.0
9 8 7 6 5 4 3 2 1 0 ppm
4.0 1.0 2.0 1.0
a b c d e f g z
OH
9b

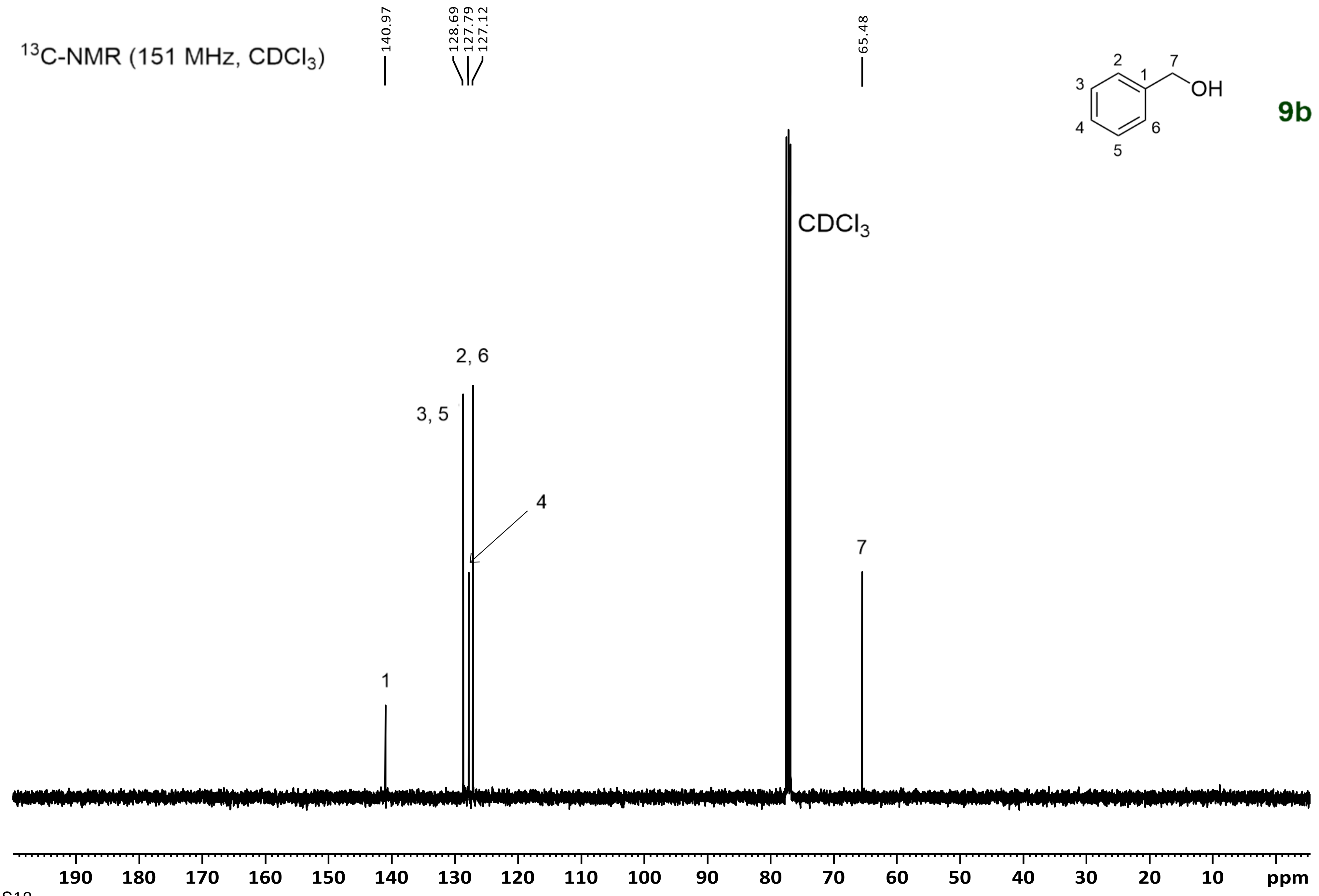

^{13}C-NMR (151 MHz, CDCl$_3$)
140.97
128.69
127.79
127.12
65.48
9b
OH
CDCl$_3$
2, 6
3, 5
4
7
1
190 180 170 160 150 140 130 120 110 100 90 80 70 60 50 40 30 20 10 ppm

Table S1: All the parameters tested during the optimization process are listed, with their corresponding isolated product yields. Methanol, 2-propanol, and water were independently evaluated as reaction solvents. In each case, the extraction process turned out to be troublesome and the product could not be isolated.

	Assessed parameters	Time (min.)	Temperature (°C)	Yield (%)
**Time test**	$CuCl_2$ 0,1 eq	10	80	82
	$CuCl_2$ 0,1 eq	15	80	82
	$CuCl_2$ 0,1 eq	30	80	65
	$CuCl_2$ 0,1 eq	60	80	60
	$CuCl_2$ 0,1 eq	80	80	57
	$CuCl_2$ 0,1 eq	90	80	48
**Reagents test**	$CuCl_2$ 0,05 eq	10	80	73
	$CuCl_2$ 0,05 eq	30	80	56
	$NaBH_4$ 3 eq.	10	80	59
	$CuCl_2$ 0,1 eq / 10 min. stirring prior to $CuCl_2$ addition	10	80	82
	$CuCl_2$ 0,05 eq / 15 min. stirring prior to $CuCl_2$ addition	10	80	71
	$CuCl_2$ 0,025 eq / 15 min. stirring prior to $CuCl_2$ addition	30	80	42
	$CuCl_2$ 0,05 eq / $NaBH_4$ and nitrostyrene order switched	10	80	68
	$CuCl_2$ 0,1 eq / $CuCl_2$ addition between $NaBH_4$ split in two portions	80	80	48
	$CuCl_2$ 0,1 eq / DETA addition	10	80	80
	$CuCl_2$ 0,05 eq / DETA addition in NaOH solution	10	80	70
	$CuCl_2$ 0,05 eq / DETA addition in NaOH solution	90	80	41
**Temp. test**	CuCl2 0,1 eq	15	60	64
	$CuCl_2$ 0,1 eq	10	110	82,6
	$CuCl_2$ 0,1 eq	o.n.	rt	79
**Solvents**	MeOH	15	80	-
	2-PrOH	15	80	-
	H_2O	15	80	-

Figure S1: ESI/MS spectrum of the crude mixture for the synthesis of **4b** after 15 minutes of stirring at 80 ˚C. The major peaks relate to the target product ($[M+H]^+$ *m/z* = 182,2) and the fragment with mass $[M+H]^+$ *m/z* = 165,0 originated from α-cleavage of **4b** [1].

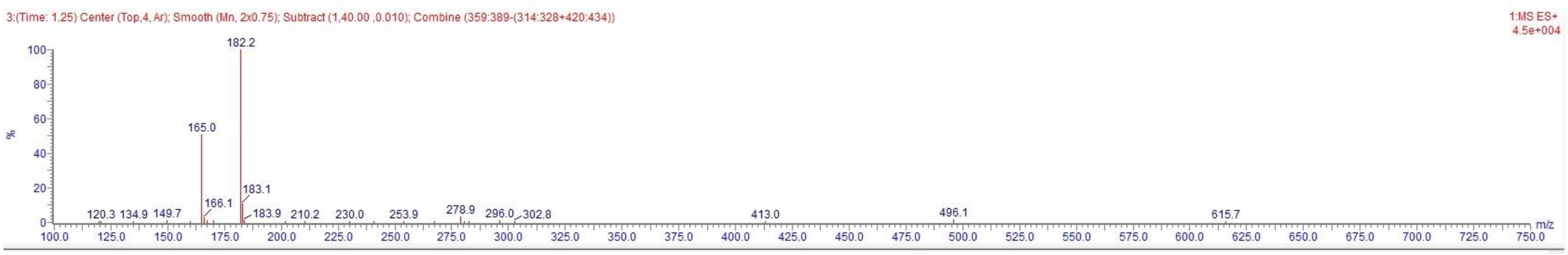

Figure S2: ESI/MS spectrum of the crude mixture for the synthesis of **4b** after 30 minutes of stirring at 80 ˚C.

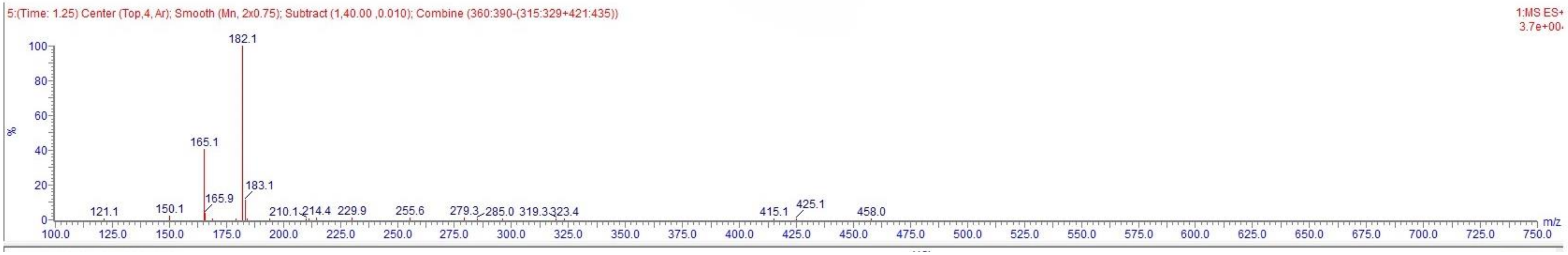

Figure S3: ESI/MS spectrum of the crude mixture for the synthesis of **4b** after 45 minutes of stirring at 80 ˚C. New structures with higher molecular masses emerge, while the concentration of **4b** decreases. The peaks with masses > 500 might indicate the formation of trimeric products.

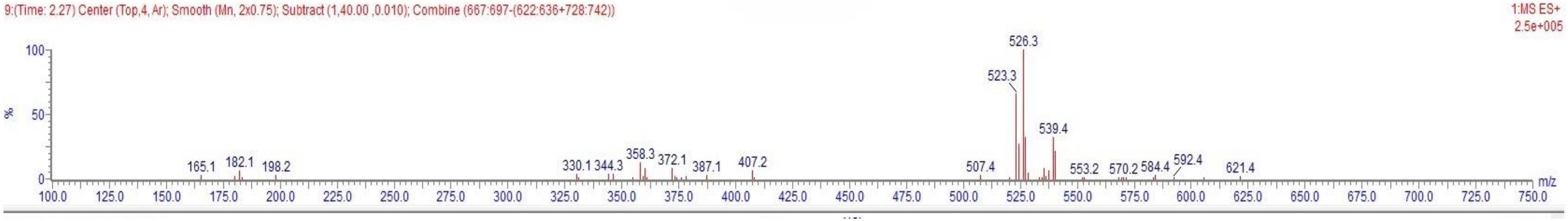

Figure S4: ESI/MS spectrum of the crude mixture for the synthesis of **4b** after 60 minutes of stirring at 80 ˚C. The peak with [M+H]$^+$ *m/z* = 151,0 is associated to the fragment originated from β-cleavage of **4b** [1]. The peak with [M+H]$^+$ *m/z* = 362,2 might be resulting from the formation of *N,N*-bis(2,5-dimethoxyphenethyl)hydroxylamine as an intermediate species.

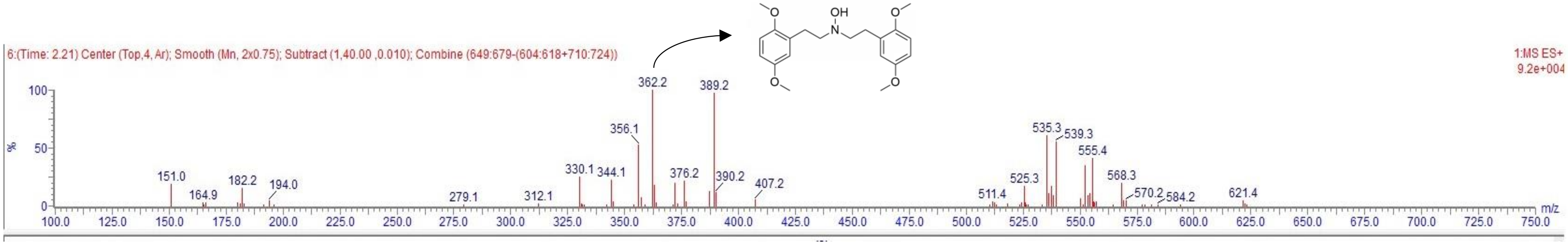

Figure S5: ESI/MS spectrum of the crude mixture for the synthesis of **4b** after 75 minutes of stirring at 80 ˚C.

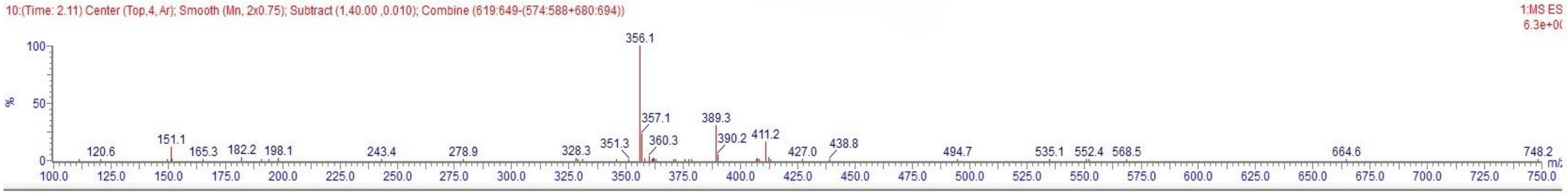

Figure S6: ESI/MS spectrum of the crude mixture for the synthesis of **4b** after 90 minutes of stirring at 80 ˚C.

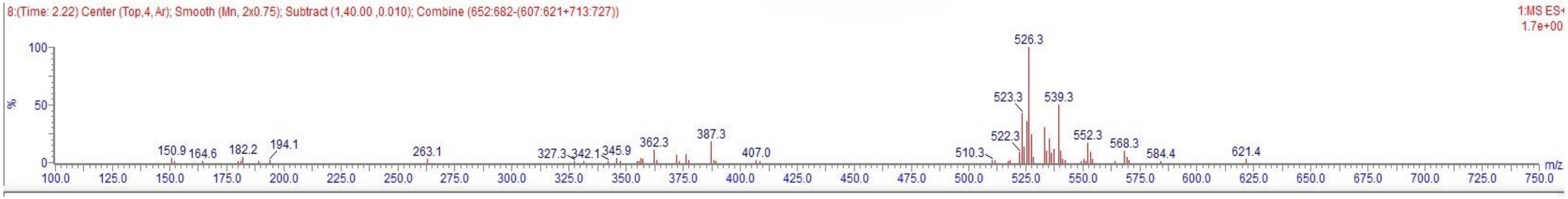

References

[1] Chen, B.H.; Liu, J.T.; Chen, H.M.; Chen, W.X.; Lin, C.H. *Applied Sciences* **2018**, *8*(7), p.1022.
